# Impacts of Sodium/Glucose Cotransporter-2 Inhibitors on Circulating Uric Acid Concentrations: A Systematic Review and Meta-Analysis

**DOI:** 10.1155/2022/7520632

**Published:** 2022-02-17

**Authors:** Abolfazl Akbari, Mahdi Rafiee, Thozhukat Sathyapalan, Amirhossein Sahebkar

**Affiliations:** ^1^Student Research Committee, Faculty of Medicine, Mashhad University of Medical Sciences, Mashhad, Iran; ^2^Department of Academic Diabetes, Endocrinology and Metabolism, Hull York Medical School, University of Hull, Hull, UK; ^3^Applied Biomedical Research Center, Mashhad University of Medical Sciences, Mashhad, Iran; ^4^Biotechnology Research Center, Pharmaceutical Technology Institute, Mashhad University of Medical Sciences, Mashhad, Iran; ^5^Department of Medical Biotechnology and Nanotechnology, Faculty of Medicine, Mashhad University of Medical Sciences, Mashhad, Iran; ^6^Department of Biotechnology, School of Pharmacy, Mashhad University of Medical Sciences, Mashhad, Iran

## Abstract

**Background:**

Several trials have assessed the antihyperglycemic effects of sodium/glucose cotransporter-2 inhibitors (SGLT2i) in patients with type 2 diabetes mellitus (T2DM). We conducted a quantitative analysis to assess the impact of SGLT2is on serum uric acid (SUA) in patients with T2DM.

**Methods:**

Placebo-controlled trials published before 13 August 2021 were identified by searching PubMed, Embase, Web of Science, and Scopus. The intervention group received SGLT2i as monotherapy or add-on treatment, and the control group received a placebo that was replaced with SGLT2i. Clinical trials providing changes in SUA were included. The mean change of SUA, glycated hemoglobin (HbA1c), fasting plasma glucose (FPG), and body weight were calculated (PROSPERO CRD42021287019).

**Results:**

After screening of 1172 papers, 59 papers were included in the systematic review. A total of 55 trials (122 groups) of 7 types of SGLT2i on patients with T2DM were eligible for meta-analysis. All SGLT2is significantly decreased SUA levels compared with the placebo groups: empagliflozin mean difference (MD) = −40.98 *μ*mol/L, 95% CI [-47.63, -34.32], dapagliflozin MD = −35.17 *μ*mol/L, 95% CI [-39.68, -30.66], canagliflozin MD = −36.27 *μ*mol/L, 95% CI [−41.62, −30.93], luseogliflozin MD = −24.269 *μ*mol/L, 95% CI [-33.31, -15.22], tofogliflozin MD = −19.47 *μ*mol/L, 95% CI [−27.40, −11.55], and ipragliflozin MD = −18.85 *μ*mol/L, 95% CI [−27.20, −10.49]. SGLT2i also decreased FPG, body weight, and HbA1c levels. SUA reduction persisted during long-term treatment with SGLT2i (except for empagliflozin), while the SUA reduction was affected by the duration of diabetes.

**Conclusions:**

SGLT2i can be a valid therapeutic strategy for patients with T2DM and comorbid hyperuricemia. Besides reducing FPG, body weight, and HbA1c, SGLT2i can significantly decrease SUA levels compared to placebo (Total MD = −34.07 *μ*mol/L, 95% CI [-37.00, -31.14]).

## 1. Introduction

Sodium/glucose cotransporter-2 inhibitors (SGLT2i) are one of the main classes of medications that are used for the management of patients with type 2 diabetes (T2DM) [1]. They also have significant renoprotective and cardioprotective properties [2–4]. These oral glucose-lowering agents have been shown to reduce the risk of cardiovascular and renal complications in patients with T2DM [2, 3, 5–7] plus several other salutary effects on autophagy pathways, neuromodulatory pathways, oxidative stress pathways, platelet function, blood pressure, and hepatic function [5, 8–12]. Dapagliflozin, canagliflozin, ipragliflozin, empagliflozin, sotagliflozin, tofogliflozin, ertugliflozin, and luseogliflozin are some of the established SGLT2is. The action of SGLT2is is independent of insulin; they reduce the renal glucose reabsorption mediated by the SGLT2 expressed along the proximal tubules [6]. Several randomized controlled trials (RCTs) with placebo-controlled groups studied the efficacy of SGLT2is in patients with and without T2DM. The change in serum uric acid (SUA) is one of the parameters which is directly or indirectly assessed in RCTs [13–72]. Increased SUA (hyperuricemia) is an important risk factor for cardiovascular and renal complications of T2DM [73, 74]. Hence, lowering SUA levels with SGLT2is could be a valid therapeutic strategy in this cohort of patients [9–12]. Di Zhao et al. [75] evaluated the effect of empagliflozin on SUA levels through a meta-analysis of clinical trials published before December 2017. They found that empagliflozin reduced serum uric acid levels and other cardiometabolic risk factors such as glycated hemoglobin (HbA1c), fasting plasma glucose (FPG), systolic and diastolic blood pressures, and body weight. Di Zhao and his team did not review other SGLT2is. A meta-analysis by Xin et al. [76] showed that SGLT2is could benefit patients with T2DM with increased SUA levels. However, this manuscript reviewed studies published before August 2017. Several recently published RCTs on the effects of SGLT2is on SUA need to be evaluated in a new meta-analysis. Moreover, limiting RCTs to placebo-controlled ones may help to identify urate-lowering properties that can be solely attributed to SGLT2i. The present study was aimed at finding any changes in SUA levels in individuals on SGLT2i based on randomized, placebo-controlled trials.

## 2. Materials and Methods

The current systematic review and meta-analysis were conducted according to the recommendations of the Preferred Reporting Items for Systematic Review and Meta-Analysis Protocols (PRISMA) [77]. This review was registered in PROSPERO (registration number: CRD42021287019).

### 2.1. Data Sources and Searches

The electronic databases of PubMed, Embase, Scopus, and Web of Science were searched to identify eligible clinical trials using relevant search terms to “Sodium-glucose cotransporter-2 inhibitors (SGLT2i)” and “ uric acid” by A.A. and M.R.; complete search strategy is available in Table S1. We identified articles published up to May 5, 2021, without restrictions on language and year of publication. In addition, we updated the article on August 13, 2021. Two authors (A.A. and M.R.) did a further manual search of the references lists of all selected papers, previous similar reviews, and pooled analysis studies to look for possible missing papers.

### 2.2. Study Selection

The two investigators (A.A. and M.R.) selected the studies according to the following criteria: (1) population: subjects (regardless of their disease) using any kind of SGLT2i; (2) intervention: SGLT2is monotherapy or as an add-on to other antidiabetic medications; (3) comparison: SGLT2is were replaced with placebo; (4) outcome: serum uric acid changes; (5) design: clinical trials; and (6) follow-up duration: at least 4 weeks. We excluded from our meta-analysis studies that were not conducted on patients with T2DM. The conference abstracts and pooled analysis studies were carefully assessed for possible duplicate data. Furthermore, several studies assessed serum uric acid at different time points. We chose the time point that was closer to 24 weeks.

### 2.3. Data Extraction and Quality Assessment

The two investigators (A.A. and M.R.) independently extracted the following data: first author, year of publication, type of study population, number of participants, demographic data, intervention (type of SGLT2i and dose regimen), follow-up duration, duration of diabetes, baseline estimated glomerular filtration rate (eGFR), and outcome (change in SUA, HbA1c, body weight, and FPG from baseline). Moreover, these authors assessed the quality of studies using the quality criteria proposed by the Joanna Briggs Institute (JBI) checklist [40]. If any disagreements existed, these were resolved through discussion or referral to another investigator (A.H.S.). Checklist questions were answered by “yes,” “no,” “unclear,” or “not/applicable.” Each “yes” answer takes 1 point. After adding up the scores, the studies were classified into three groups based on their risk of bias: high risk of bias (scores between 0 and 5), intermediate-risk (scores between 6 and 10), and low-risk groups (scores between 11 and 13).

### 2.4. Publication Bias and Statistical Analysis

Publication bias was examined using funnel plots, Egger's test and Begg's test. Mean differences (MD) and 95% confidence interval (CI) in SUA levels were calculated using a random-effects model to evaluate the effects of SGLT2is on SUA, HbA1c, body weight, and FPG. Heterogeneity was calculated using *I*^2^, with *I*^2^ values >50% representing moderate heterogeneity. *P*-value less than 0.05 was considered as statistically significant for the outcome and heterogeneity analyses. Random-effect meta-regression analysis was done to assess the effects of the patient's duration of diabetes, treatment period, and SGLT2i dosage on SUA level changes. Data analysis was done using the Comprehensive Meta-Analysis software (CMA) V.3.

## 3. Results

**Figure 1:**
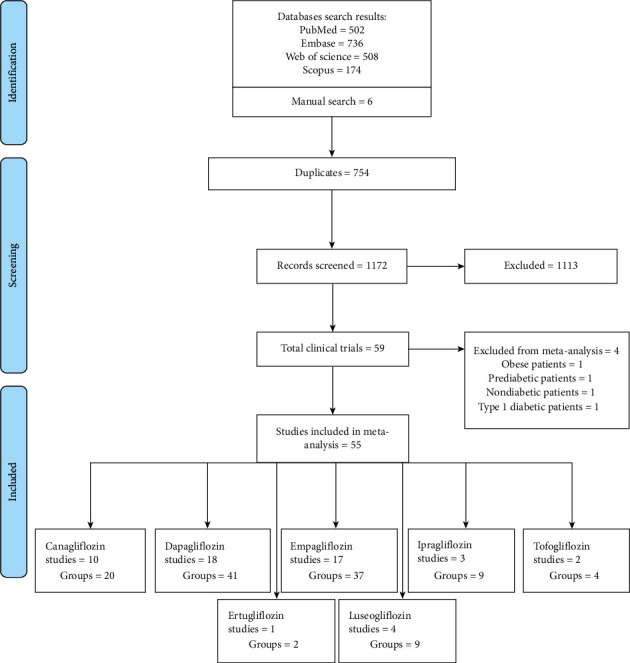
PRISMA flow diagram.

A total of 1920 papers were collected during the initial electronic search. Through a manual search, six papers were identified. Among those papers, 754 were duplicates, so the 1172 remaining papers were assessed for eligibility criteria. Finally, 59 trials met the inclusion criteria, and 55 trials were included in the meta-analysis. The screening, assessing, and analyzing steps are shown in Figure 1. Seven types of SGLT2is were assessed, including canagliflozin, dapagliflozin, empagliflozin, ipragliflozin, tofogliflozin, ertugliflozin, and luseogliflozin. Descriptive characteristics of the 59 included trials (9 types of SGLT2is) are presented in Table 1.

### 3.1. Outcome

**Figure 2:**
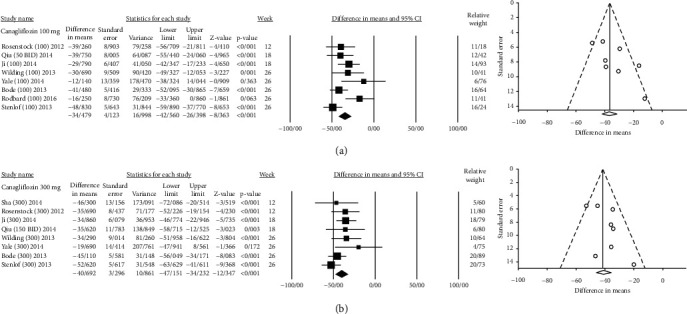
Mean difference and 95% confidence intervals for changes in serum uric acid level for canagliflozin compared to placebo ((a) canagliflozin 100 mg; and (b) canagliflozin 300 mg).

**Figure 3:**
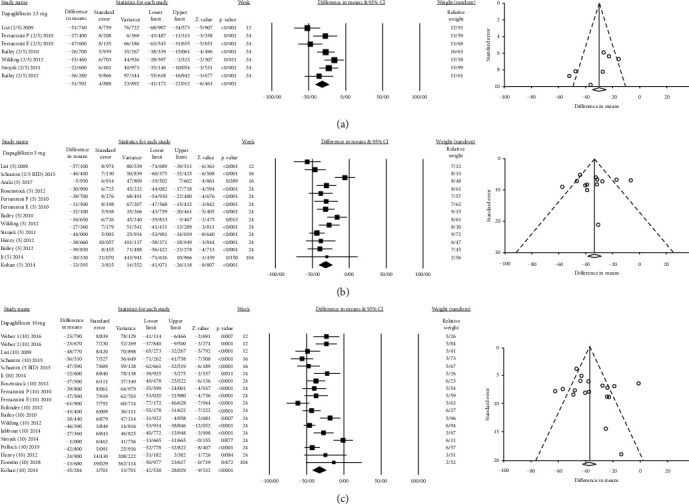
Mean difference and 95% confidence intervals for changes in serum uric acid level for dapagliflozin compared to placebo ((a) dapagliflozin 2.5 mg; (b) dapagliflozin 5 mg; and (c) dapagliflozin 10 mg).

**Figure 4:**
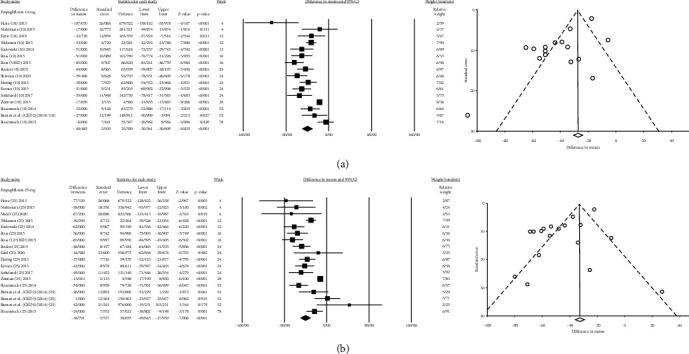
Mean difference and 95% confidence intervals for changes in serum uric acid level for empagliflozin compared to placebo ((a) empagliflozin 10 mg; and (b) empagliflozin 25 mg).

**Figure 5:**
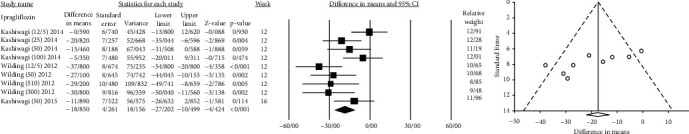
Mean difference and 95% confidence intervals for changes in serum uric acid level for ipragliflozin (range of 12.5 mg to 300 mg) compared to placebo.

**Figure 6:**
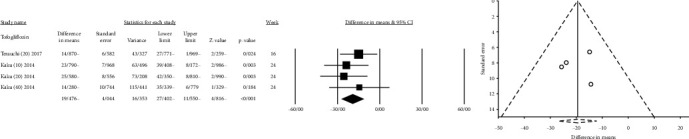
Mean difference and 95% confidence intervals for changes in serum uric acid level for tofogliflozin (range of 10 mg to 40 mg) compared to placebo.

**Figure 7:**
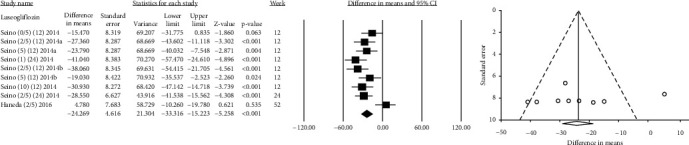
Mean difference and 95% confidence intervals for changes in serum uric acid level for luseogliflozin (range of 0.5 mg to 10 mg) compared to placebo.

Of the 36,215 patients, 23,494 received different SGLT2is in different dosages versus 12,721 patients who received placebo. The effect size, population, and heterogeneity of SGLT2is included in meta-analysis are shown in Table 2. SGLT2is considerably decreased SUA levels compared with placebo (Total MD, -34.07 *μ*mol/L, 95% CI [-37.00, -31.14], empagliflozin MD, -40.98 *μ*mol/L, 95% CI [-47.63, -34.32], dapagliflozin MD, -35.17 *μ*mol/L, 95% CI [-39.68, -30.66], canagliflozin MD, −36.27 *μ*mol/L, 95% CI [−41.62, −30.93], luseogliflozin MD, -24.269 *μ*mol/L, 95% CI [-33.31, -15.22], tofogliflozin MD, -19.47 *μ*mol/L, 95% CI [−27.40, −11.55], and ipragliflozin MD, -18.85 *μ*mol/L, 95% CI [−27.20, −10.49]) (Figures S1–S3 and Figures 2–7).

Out of 122 comparisons between the different dosages of SGLT2is and placebo, 21 comparisons showed that SGLT2is did not significantly reduce the SUA. After the removal of studies which were conducted only on patients with chronic kidney disease (CKD) [22, 43, 68, 72], the MD of SUA changes of dapagliflozin compared to placebo increased to -36.29 *μ*mol/L (95% CI [-40.53, -32.05], *I*^2^ = 69.3%), the MD of SUA changes of canagliflozin compared to placebo increased to -37.44 *μ*mol/L (95% CI [-42.90, -31.97], *I*^2^ = 68.0%), and MD of SUA changes of empagliflozin compared to placebo increased to -43.79 *μ*mol/L (95% CI [-50.75, -36.83], *I*^2^ = 85.9%).

#### 3.1.1. Canagliflozin

Ten clinical trials evaluated the effect of canagliflozin (range of 50 mg to 600 mg) on SUA. Canagliflozin 300 mg reduced the SUA, FPG, body weight, and HbA1c more than canagliflozin 100 mg (Table 2). Moreover, the results of metaregression, shown in Table 3, demonstrated that the amount of SUA change was not significantly correlated with dosage and weeks of treatment. However, SUA change was positively correlated with the duration of diabetes (Coefficient = 1.581 [0.148, 3.014]; *P* = 0.03) (Table 3). Figure S4 shows the scatter plots of metaregression by week, SGLT2i dosage, and duration of diabetes covariates. Figures S7, S10, and S13 show the forest plot of HbA1c, FPG, and body weight changes, respectively.

#### 3.1.2. Dapagliflozin

Eighteen clinical trials with a total of 41 comparisons examined the effect of dapagliflozin (range of 1 mg to 50 mg) on SUA levels. The pooled effects of different doses of dapagliflozin on SUA, HbA1c, and FPG are reported in Table 2. MD of HbA1c, body weight, and FPG changes was lower in dapagliflozin studies than other types of SGLT2i. Furthermore, the results of random-effects meta-regression indicated that the amount of SUA change does not correlate with dosage or weeks of treatment, but SUA change was positively correlated with the duration of diabetes (Coefficient = 1.906 [1.218, 2.594]; *P* < 0.001) (Table 3). Figure S5 shows the scatter plots of metaregression by week, dosage, and duration of diabetes covariates. Figures S8, S11, and S14 show the forest plot of HbA1c, FPG, and body weight changes, respectively. Moreover, one study was removed because it was conducted on prediabetic patients. The findings showed that dapagliflozin reduced SUA levels (MD = −62 ± 47 *μ*mol/L) [45].

#### 3.1.3. Empagliflozin

Seventeen trials assessed the effect of empagliflozin (range of 5 mg to 100 mg) on SUA. Empagliflozin had the highest rate of SUA reduction (MD = −40.98; CI [-47.63, -34.32]; *I*^2^ = 84.9%). Empagliflozin effects on SUA, HbA1c, body weight, and FPG are shown in Table 2. Scatter plots of metaregression by the week of treatment and dosage covariates are shown in Figure S6. Figures S9, S12, and S15 show the forest plot of HbA1c, FPG, and body weight changes, respectively. We removed the Zanchi et al. study from the meta-analysis. They employed nondiabetic patients to measure the effect of empagliflozin 10 mg; the results also showed a reduction in SUA (MD = −97 ± 36 *μ*mol/L) [70].

#### 3.1.4. Other SGLT2i

The effects of other SGLT2is on SUA, HbA1c, body weight, and FPG are also reported in Table 2. Three studies assessed ipragliflozin (range of 12.5 mg to 300 mg), two studies assessed tofogliflozin (range of 10 mg to 40 mg), and four studies assessed luseogliflozin (range of 0.5 mg to 10 mg) effects on SUA levels. Four studies were removed from the meta-analysis because they did not assess patients with T2DM. A recent study in 2020 assessed the effects of 12 weeks of treatment with licogliflozin on 123 obese patients. MD of SUA change in different doses was between -65.1 and -74.4 *μ*mol/L [69]. Van Raalte et al. in 2019 assessed a 24-week treatment with sotagliflozin 200 mg or 400 mg on 955 type 1 diabetes patients compared with 479 patients in the placebo group; MD of SUA was calculated −32.71 ± 38.95 *μ*mol/L [63].

### 3.2. Publication Bias

Regarding Egger's test, canagliflozin 100 mg, canagliflozin 300 mg, empagliflozin 10 mg, and empagliflozin 25 mg had publication bias (*P* − value < 0.05). However, Begg's test did not show any publication bias, except for canagliflozin (total).

## 4. Discussion

The current meta-analysis of 55 placebo-controlled trials analyzed the data of 23,494 patients who received SGLT2is compared with 12,721 patients who received a placebo. The mean difference of SUA changes was about -34.07 *μ*mol/L (95% CI [-37.00, -31.14], *I*^2^ = 78.8%) among T2DM patients. Empagliflozin showed more potential in SUA reduction than other SGLT2is, while ipragliflozin had the least SUA changes.

There are six meta-analyses on this topic, two of which focused specifically on SUA change. Wu et al. assessed the impact of SGLT2i as an add-on treatment to insulin therapy compared to the control group in patients with T2DM, which received a placebo in addition to insulin. They calculated MD of SUA change -26.16 *μ*mol/L (95% CI [-42.14, -10.17], *I*^2^ = 80%) through assessment of 5 comparisons [78]. Yumo Zhao et al. specifically focused on SUA changes of 62 trials, which compared the effects of SGLT2is with placebo or active control or standard care. Overall MD of SUA changes was -37.73 *μ*mol/L (CI [-40.51, -34.95], *I*^2^ = 73.5%) [79].

Dapagliflozin was studied more than the other SGLT2is. In accordance with our study, a previous meta-analysis on 4454 patients showed that dapagliflozin can significantly reduce SUA; the weighted mean difference (WMD) of SUA changes was about -41.50 *μ*mol/L (95% CI [-47.22, -35.79]; *I*^2^ = 50%), while it was about -35.17 *μ*mol/L (95% CI [-39.68, -30.66], *I*^2^ = 73.9%) in our study [80]. The effects of dapagliflozin on SUA were also assessed by Zhang et al. on 5302 patients, with results being similar to those of our study (WMD -36.17 *μ*mol/L; 95% CI: -40.99, -31.36; *I*^2^ = 64%) [81]. Both studies compared dapagliflozin with a placebo.

In agreement with us, Xin et al. assessed SUA changes obtained with 5 types of SGLT2i compared with placebo or control or standard care. All SGLT2is significantly decreased SUA levels compared with placebo; canagliflozin WMD –37.02 *μ*mol/L (95% CI [–38.41, -35.63]), dapagliflozin WMD –38.05 *μ*mol/L (95% CI [–44.47, -31.62]), empagliflozin WMD –42.07 *μ*mol/L (95% CI [–46.27, –37.86]), tofogliflozin WMD -18.97 *μ*mol/L (95% CI [-28.79, -9.16]), and ipragliflozin WMD -19.75 *μ*mol/L (95% CI [-28.17, -11.34]) [76]. Furthermore, Yumo Zhao et al. performed a metaregression and concluded that only the effect of dapagliflozin depended on the administration dosage. In addition, the metaregression of Yumo Zhao et al. showed that the reduction of SUA could be persistent with long-term, 104-week administration of SGLT2is [79]. Conversely, our study showed no relationship between SUA reduction and duration (except for empagliflozin) and dosage of SGLT2i. However, our data showed that SUA was reduced more in the canagliflozin and dapagliflozin groups, with a more pronounced reduction observed in patients with a longer duration of diabetes. Perhaps, longer duration of diabetes may alter the expression of SGLT2, glucose transporter 9 (GLUT9), or related unknown pathways in the kidney, thus favouring uric acid excretion. Di Zhao et al. specifically reviewed the effect of empagliflozin on some cardiometabolic risk factors [75]. In accordance with our review, they showed that empagliflozin could significantly reduce SUA level, HbA1c, and FPG. However, there are some differences: the mean change of HbA1c and FPG, unlike SUA, was higher in their study. The differences may be due to the mean treatment period, the number of patients, and different analysis tools.

Increased SUA causes inflammation in adipocytes as well as endothelial dysfunction, which reduces nitric oxide bioavailability and leads to insulin resistance. Moreover, uric acid impairs glucose uptake in skeletal muscle, which reduces insulin-stimulated glucose uptake [82]. Insulin resistance leads to hyperinsulinemia, which elevates SUA through lowering renal uric acid excretion [83, 84].

SGLT2is could significantly decrease SUA through several mechanisms. GLUT9 protein is expressed in two subtypes, namely, GLUT9a and GLUT9b, localized in the apical and basolateral membrane of the proximal tubule, respectively. GLUT9 subtypes regulate uric acid transportation and concentration [85]. Chino et al. revealed that the urinary excretion rate of uric acid strongly correlated with the urinary glucose excretion, demonstrating the relation between SUA and glycosuria [86]. Raised glucose concentration resulting from SGLT2i administration could also disturb the reabsorption of uric acid in the proximal tubule through GLUT9b [87]. After removing the studies that were conducted on CKD patients, the SUA reduction was increased, which is consistent with the proposed model for uricosuric effects of SGLT2i by Chino et al. [86]. Hence, SGLT2i induce more pronounced glycosuria in the presence of higher eGFR values. Moreover, part of the SUA reduction can be explained by the body weight loss induced by SGLT2is. Previous studies showed a strong positive correlation between body mass index and SUA levels [88–91]. Body weight loss is recommended for the management of gout [92, 93]. A possible explanation is that insulin resistance increases the reabsorption of organic anions like urate [94].

### 4.1. Limitations and Strengths

Our study has some limitations. First, due to the paucity of available studies, we could not perform a meta-analysis for ertugliflozin and sotagliflozin. Second, some studies did not report the standard deviation or related data to calculate it. Third, trials with CKD patients, whose plasma UA level may be increased because of disease deterioration, could interfere with the results. Fourth, some studies had some dropouts, but they reported baseline data of all patients. Fifth, the baseline SUA level and follow-up period were different across the studies. Sixth, some of the administered doses of SGLT2i were not within the approved dose range for the T2DM treatment. Finally, the heterogeneity of SUA data was moderate or high except for canagliflozin (300 mg) and tofogliflozin. The comparison with active control groups and paucity of available studies were the other limitations of previous meta-analyses.

### 4.2. Conclusion

All SGLT2i analyzed in the meta-analysis can reduce SUA in patients with T2DM (MD = −34.076; CI [-37.006, -31.146]). The ability to reduce SUA is one of the advantages of SGLT2is over other antidiabetic medications, particularly in patients with T2DM and comorbid hyperuricemia. Moreover, the urate-lowering properties exerted by SGLT2i may partly explain their well-established renoprotective and cardioprotective actions. More placebo-controlled studies are warranted for luseogliflozin, licogliflozin, sotagliflozin, and ertugliflozin to clarify their effects on SUA.

## Figures and Tables

**Table 1:** Characteristics of included studies.

Author	Size	Females (%)	Dosage (mg)	Treatment duration	SUA I (mg/dL)†	SUA P (mg/dL)†	HbA1c I (%)†	HbA1c P (%)†	FPG I (mg/dL)†	FPG P (mg/dL)†	Duration of diabetes (*Y) I*	Duration of diabetes (*Y*) *P*	eGFR (ml/min/1.73 m^2^) I†	eGFR (ml/min/1.73 m^2^) P†	Risk of bias
*Canagliflozin*															

Rosenstock et al.∗ [48]	386	188 (48.7)	50 100 200 300 300 BID	12 weeks	—	—	8.01 7.81 7.57 7.70 7.71	7.71	171.0 167.4 158.4 158.4 154.8	162.0	5.6 6.1 6.4 5.9 5.8	6.4	—	—	Moderate

Ji et al. [28]	636	314 (46.4)	100 300	18 weeks	315.7 323.3	322.6	8.0 ± 0.9 8.0 ± 0.9	7.9 ± 0.9	156.5 ± 33.6 160.2 ± 36.0	158.2 ± 32.7	6.8 6.9	6.4	93.9		Low

Sha et al. [56]	35	5 (13.9)	300	12 weeks	352.6	339.7	7.6 ± 0.5	7.7 ± 0.6	154.95 ± 1.1	147.74 ± 1.4	8.6	8.4	90.8	103.8	Moderate

Wilding et al. [66]	306	230 (49.0)	100 300	26 weeks	322.3 340.1	332.9	8.1 ± 0.9 8.1 ± 0.9	8.1 ± 0.9	172.97 ± 2.3 167.56 ± 2.1	9.4 ± 2.2	9 9.4	10.3	91.0 91.9	87.4	Low

Stenlöf et al. [59]	584	326 (55.8)	100 300	26 weeks	320.0 326.3	333.1	8.0 ± 1.0 8.0 ± 1.0	8.0 ± 1.0	172.97 ± 2.4 172.97 ± 2.4	167.56 ± 2.1	4.5 4.3	4.2	—	—	Moderate

Yale et al. [68]	211	106 (39.4)	100 300	26 weeks	433.7 442.2	433.4	7.9 ± 0.9 8.0 ± 0.8	8.0 ± 0.9	169.36 ± 2.6 158.55 ± 3.2	160.8 ± 2.4	15.6 17	16.4	39.7 38.5	40.1	Moderate

Rodbard et al. [46]	171	92 (43.2)	100	26 weeks	318.3	336.7	8.5 ± 0.9	8.4 ± 0.8	185.5 ± 46.2	180.4 ± 37.8	9.8	10.1	91.4	89.6	Moderate

Bode et al. [18]	584	318 (44.5)	100 300	26 weeks	339.1 341.4	343.4	7.8 ± 0.8 7.7 ± 0.8	7.8 ± 0.8	160.4 ± 38.7 153.2 ± 36.6	156.8 ± 38.9	12.3 11.3	11.4	77.6 78.7	76.1	Moderate

CANVAS∗ [38]	10142	3629 (36)	100/300	26 weeks	350.93 ± 95.17	350.93 ± 95.17	8.2 ± 0.9	8.2 ± 0.9	—	—	13.7	13.5	76.7	76.2	Low

Qiu et al. [44]	239	149 (53.4)	50 BID 150 BID	18 weeks	310.7 323.8	322.8	7.6 ± 0.9 7.6 ± 0.9	7.7 ± 0.9	9.0 ± 2.0 9.1 ± 1.9	9.0 ± 1.9	6.7 7.3	7	86.9 85.9	84.8	Low

*Dapagliflozin*															

Weber et al. [65]	402	202 (45.0)	10	12 weeks	334 · 95 ± 92 · 59	325 · 28 ± 78 · 92	8 · 1 ± 0 · 9	8 · 0 ± 1 · 0	162.16 ± 45.04	160.36 ± 43.24	7.7	7.3	84.8	87.0	Moderate

Ji et al.∗ [29]	338	136 (34.6)	5 10	24 weeks	309.3 ± 71.38 297.4 ± 77.32	321.19 ± 95.17	8.14 ± 0.74 8.28 ± 0.95	8.35 ± 0.95	154.37 ± 31.68 162.22 ± 43.30	167.13 ± 42.79	1.15 1.67	1.30	91.6 91.7	94.1	Moderate

Jabbour et al.∗ [27]	292	202 (45.1)	10	24 weeks	—	—	7.9 ± 0.8	8.0 ± 0.8	162.2 ± 36.8	163.0 ± 34.5	5.70	5.64	—	—	Moderate

Bailey et al. [14]	282	141 (50.0)	1 2.5 5	24 weeks	321.19 ± 78.22 317.62 ± 87.50 308.70 ± 92.43	339.04 ± 96.89	7.8 ± 0.98 8.1 ± 1.07 7.9 ± 1.03	7.8 ± 1.12	155.49 ± 48.28 159.81 ± 51.53 157.11 ± 41.62	161/62 ± 57.47	1.6 1.5 1.4	1.1	—	—	Moderate

Ferrannini et al.∗ [21]	485	256 (52.8)	2.5 P 2.5 E 5 P 5 E 10 P 10 E	24 weeks	—	—	7.92 ± 0.90 7.86 ± 0.94 8.01 ± 0.96 7.99 ± 0.99 7.82 ± 0.91 7.99 ± 1.05	7.84 ± 0.87	164.1 ± 48.0 162.2 ± 45.0 ±166.6 41.5 160.6 ± 45.9 157.0 ± 50.9 168.1 ± 57.9	159.9 ± 42.1	0.50 0.20 0.25 0.50 0.45 0.40	0.50	—	—	Moderate

Henry et al. [26]	809	438 (53.8)	5 10	24 weeks	293.9 ± 91.0 307.5 ± 87.4	301.6 ± 85.1 302.8 ± 77.3	9.21 ± 1.31 9.10 ± 1.28	9.14 ± 1.32 9.03 ± 1.30	193.87 ± 56.21 189.54 ± 58.01	197.11 ± 60.36 190.45 ± 54.05	1.6 2.2	1.6	—	—	Moderate

Strojek et al.∗ [60]	596	307 (51.5)	2.5 5 10	24 weeks	301.6 ± 81.2 303.9 ± 79.8 301.0 ± 82.4	315.2 ± 93.6	8.11 ± 0.75 8.12 ± 0.78 8.07 ± 0.79	8.15 ± 0.74	172.25 ± 38.37 174.41 ± 38.19 172.07 ± 36.75	172.61 ± 37.29	7.7 7.4 7.2	7.4	80.9 83.5 82.2	80.2	Moderate
Bailey et al. [16]	541	254 (46.5)	2.5 5 10	24 weeks	322.4 ± 80.4 323.0 ± 88.3 323.0 ± 79.9	314.1 ± 79.0	7.99 ± 0.90 8.17 ± 0.96 7.92 ± 0.82	8.11 ± 0.96	161.44 ± 43.06 169.18 ± 49.00 156.03 ± 38.73	165.58 ± 46.30	6.0 6.4 6.1	5·8	—	—	Low

Ramirez-Rodriguez et al. [45]	24	17 (70.8)	10	12 weeks	334 ± 70	312 ± 101	—	—	106.3 ± 7.2	108.1 ± 7.2	—	—	—	—	Low

Weber et al. [64]	557	263 (42.9)	10	12 weeks	321.19 ± 83.27	321.19 ± 77.32	8.1 ± 1.0	8.0 ± 0.9	142.2 ± 40.5	163.1 ± 44.1	8.2	7.6	85.1	86.7	Moderate

Bolinder et al.∗ [19]	167	80 (40.5)	10	24 weeks	346.8 ± 68.9	338.4 ± 61.7	7.19 ± 0.44	7.16 ± 0.53	148.0 ± 24.7	149.6 ± 25.1	6.0	5.5	—	—	Moderate

Rosenstock et al. [51]	420	212 (50.5)	5 10	24 weeks	—	—	8.40 ± 1.03 8.37 ± 0.96	8.34 ± 1.00	168.6 ± 52.1 164.9 ± 46.3	160.7 ± 47.0	5.64 5.75	5.07	—	—	Moderate

Fioretto et al. [22]	320	159 (49.7)	10	24 weeks	—	—	8.33 ± 1.08	8.03 ± 1.08	181/98 ± 66.66	172.97 ± 54.05	14.3	14.5	53.3	53.6	Moderate

Wilding et al.∗ [67]	660	418 (52.2)	2.5 5 10	24 weeks	326.0 ± 89.2 323.6 ± 95.2 324.8 ± 93.9	333.7 ± 98.3	8.46 ± 0.78 8.62 ± 0.89 8.57 ± 0.82	8.47 ± 0.77	180.1 ± 59.9 185.4 ± 58.7 173.1 ± 54.9	170.6 ± 57.2	13.6 13.1 14.2	13.5	78.5 76.2 79.3	79.9	Low

Kohan et al. [36]	252	88 (34.9)	5 10	104 weeks	434.2 ± 126.1 424.09 ± 101.71	419.33 ± 115.39	8.30 ± 1.04 8.22 ± 0.97	8.53 ± 1.29	161 ± 56 165 ± 67	150 ± 48	16.9 18.2	15.7	44.2 43.9	45.6	Moderate

List et al. [39]	333	164 (49.2)	2.5 5 10 20 50	12 weeks	327.14 ± 71.38 309.3 ± 77.32 327.14 ± 71.38 315.24 ± 77.32 333.09 ± 83.27	327.14 ± 83.27	7.6 ± 0.7 8.0 ± 0.9 8.0 ± 0.8 7.7 ± 0.9 7.8 ± 1.0	7.9 ± 0.9	145 ± 34 153 ± 48 148 ± 38 149 ± 41 153 ± 42	150 ± 46	—	—	—	—	Moderate

Pollock et al. [43]	291	86 (29.6)	10	24 weeks	399 · 4 ± 98 · 9 424 · 9 ± 102 · 8	414 · 9 ± 92 · 6	8 · 44 ± 1 · 0 8 · 20 ± 1 · 0	8 · 57 ± 1 · 2	—	—	17.55	17.71	50.2	47.7	Moderate

Schumm -Draeger et al. [53]	401	220 (55.1)	2.5 BID 5 BID 10	16 weeks	337.87 ± 72.57 331.93 ± 81.96 349.77 ± 89.81	337.28 ± 87.50	7.77 ± 0.75 7.78 ± 0.76 7.71 ± 0.71	7.94 ± 0.85	153.33 ± 33.33 155.31 ± 31.89 155.31 ± 36.39	157.83 ± 35.85	4.80 5.12 5.45	5.53	—	—	Low

Araki et al. [13]	173	53 (28.9)	5	16 weeks	321.19 ± 77.32	315.24 ± 83.27	8.3 ± 0.8	8.5 ± 0.9	160.7 ± 44.9	159.7 ± 38.0	15.3	14.2	—	—	Moderate

*Empagliflozin*															

Zanchi et al. [70]	45	18 (40.0)	10	4 weeks	303 ± 70	275 ± 73	5.4 ± 0.3	5.4 ± 0.3	90 ± 12.61	90 ± 7.20	—	—	112.9	113.1	Moderate

Shimizu et al.∗ [57]	96	19 (19.8)	10	24 weeks	344.98 ± 83.27	339.04 ± 89.22	6.82 ± 1.00	6.89 ± 0.92	—	—	3.19	2.7	66.1	64.6	Moderate

Ross et al. [52]	965	445 (46.1)	10 25 12.5 BID 5 BID	16 weeks	316 ± 126 328 ± 122 316 ± 120 327 ± 131	330 ± 115	7.78 ± 0.79 7.73 ± 0.79 7.79 ± 0.88 7.84 ± 0.75	7.69 ± 0.72	156.75 ± 37.83 158.55 ± 32.43 162.16 ± 39.63 162.16 ± 41.44	160.36 ± 34.23	—	—	89.5 88.9 88.6 89.7	89.5	Moderate

Kovacs et al. [37]	265	257 (51.6)	10 25	76 weeks	288 ± 116 271 ± 117	275 ± 113	8.07 ± 0.898.06 ± 0.82	8.16 ± 0.92	151.35 ± 37.83 151.35 ± 37.83	151.35 ± 39.63	—	—	84.3 87.4	85.5	Moderate

Haring et al. [24]	671	276 (43)	10 25	24 weeks	314 ± 127 298 ± 115	307 ± 110	8.07 ± 0.81 8.10 ± 0.83	8.15 ± 0.83	150.99 ± 32.79 156/75 ± 33.69	151.71 ± 35.85	—	—	86.5 88.3	86.9	Moderate
Kahl et al. [31]	84	26 (30.9)	25	24 weeks	365.26	381.84	6.8 ± 0.5	6.7 ± 0.7	135.13 ± 25.22	129.72 ± 23.42	3	3.33	—	—	Low

Kadowaki et al. [30]	547	137 (25)	5 10 25 50	12 weeks	283 ± 120 277 ± 124 277 ± 101 262 ± 136	271 ± 127	7.92 ± 0.707.93 ± 0.717.93 ± 0.788.02 ± 0.65	7.94 ± 0.74	154.0 ± 27.9156.8 ± 28.5156.0 ± 29.9158.0 ± 28.4	156.3 ± 28.9	—	—	86.5 85.8 85.2 86.5	84.6	Moderate

Tikkanen et al. [62]	723	328 (39.9)	10 25	12 weeks	341.85 ± 81.78 338.27 ± 79.52	347.37 ± 82.73	7.87 ± 0.77 7.92 ± 0.72	7.90 ± 0.72	156.75 ± 37.83 162.16 ± 37.83	160.36 ± 36.03	—	—	83.4 83.5	85.0	Moderate

Zinman et al. [71]	6652	2004 (28.5)	10 25	28 weeks	350.93	356.88	—	—	—	—	—	—	—	—	Moderate

Rosenstock et al. [50]	494	218 (44)	10 25	78 weeks	313 ± 102 335 ± 119	327 ± 122	8.3 ± 0.1 8.3 ± 0.1	8.1 ± 0.1	138.73 145.94	142.34	—	—	85 83	84	Moderate

Rosenstock et al. [49]	563	307 (55)	10 25	52 weeks	326 ± 127 331 ± 123	326 ± 121	8.39 ± 0.05 8.29 ± 0.05	8.33 ± 0.05	159.09 150.27	151.35	—	—	—	—	Moderate

Mordi et al. [41]	23	6 (26.1)	25	6 weeks	—	—	—	—	—	—	8.7	8.7	—	—	Moderate

Roden et al. [95]	676	266 (39.3)	10 25	76 weeks	293 ± 109	307 ± 133	7.87 ± 0.88	7.91 ± 0.78	153.15 ± 32.43	154.95 ± 36.03	—	—	87.7 87.5	86.8	Moderate

Heise et al. [25]	78	11 (14.1)	10 25 100	4 weeks	—	—	7.2 ± 0.7 7.5 ± 0.8 7.1 ± 0.9	6.9 ± 0.9	186.2 ± 93.0 167.5 ± 39.6 150.0 ± 31.7	153.9 ± 40.5	6.5 5.8 6.3	6.9	—	—	Moderate

Kario et al.∗ [33]	131	62 (47.6)	10	12 weeks	322.98 ± 89.22	318.81 ± 89.22	6.6 ± 0.8	6.6 ± 0.8	—	—	10.6	9.6	69.2	69.6	Moderate

Nishimura et al. [42]	60	13 (21.6)	10 25	4 weeks	265 ± 155 268 ± 84	304 ± 147	7.99 ± 0.837.73 ± 0.75	8.00 ± 0.82	151.0 ± 21.6 151.9 ± 23.3	154.5 ± 19.8	—	—	76.5 80.7	82.6	Moderate

Søfteland et al. [58]	327	130 (39.7)	10 25	24 weeks	301 ± 124 297 ± 116	310 ± 118	7.97 ± 0.84 7.97 ± 0.82	7.97 ± 0.85	9.3 ± 2.2 9.4 ± 2.3	9.1 ± 1.8	—	—	90.4 93.4	93.0	Moderate

Barnett et al. [72] (CKD2)	292	113 (39.0)	10 25	52 weeks	341 ± 126 337 ± 159	339 ± 125	8 · 02 ± 0 · 84 7 · 96 ± 0 · 73	8.09 ± 0.80	145.94 ± 34.23 147.74 ± 34.23	144.14 ± 37.82	—	—	70·8 72·3	71·8	Low

Barnett et al. [72] (CKD3)	375	161 (43.0)	25	52 weeks	419 ± 158	439 ± 153	8 · 02 ± 0 · 84	8 · 09 ± 0 · 80	142.34 ± 36.03	144.14 ± 5.45	—	—	44·3	45·4	Low

Barnett et al. [72] (CKD4)	74	34 (45.9)	25	52 weeks	559 ± 126	583 ± 162	8 · 06 ± 1 · 05	8 · 16 ± 0 · 99	156.75 ± 54.05	147.74 ± 57.65	—	—	24.4	22.0	Low

*Ipragliflozin*															

Kashiwagi et al.∗ [35]	331	126 (35.0)	12.5 25 50 100	12 weeks	—	—	8.39 ± 0.90 8.32 ± 0.83 8.33 ± 0.80 8.25 ± 0.76	8.36 ± 0.79	185.4 ± 40.0 178.0 ± 38.8 173.4 ± 34.9 177.5 ± 32.1	186.4 ± 39.5	6.3 6.4 6.6 7.8	6.3	—	—	Moderate

Kashiwagi et al.∗ [34]	129	39 (30.2)	50	16 weeks	289.07 ± 65.43	272.42 ± 73.16	8.40 ± 0.86	8.25 ± 0.68	175.9 ± 42.6	174.1 ± 39.1	5.9	7.5	87.80	86.11	Moderate

Wilding et al.∗ [66]	304	167 (48.8)	12.5 50 150 300	12 weeks	—	—	7.78 ± 0.64 7.76 ± 0.66 7.73 ± 0.69 7.87 ± 0.82	7.68 ± 0.60	158.55 ± 41.44 153.15 ± 36.03 151.35 ± 30.63 156/75 ± 37.83	154.95 ± 27.02	6.8 6.0 5.7 5.5	5.7	—	—	Moderate
*Tofogliflozin*															

Terauchi et al.∗ [61]	201	63 (31.3)	20	16 weeks	300.37 ± 74.35	311.08 ± 84.46	8.53 ± 0.75	8.40 ± 0.65	163.4 ± 47.5	162.4 ± 43.2	15.02	9.36	79.7	79.5	Low

Kaku et al.∗ [32]	212	76 (33.2)	10 20 40	24 weeks	283.72 ± 60.07 297.99 ± 70.78 305.73 ± 75.54	302.75 ± 82.68	8.45 ± 0.75 8.34 ± 0.81 8.37 ± 0.77	8.41 ± 0.78	170.2 ± 32.4 168.7 ± 29.6 167.9 ± 37.0	168.8 ± 24.9	6.3 6.4 6.7	6.0	84.90 86.78 86.00	83.78	Moderate

*Luseogliflozin*															

Haneda et al. [23]	145	34 (23.4)	2.5	24 weeks	350.93 ± 78.51	375.32 ± 85.06	7.72 ± 0.68	7.69 ± 0.65	140.4 ± 30.2	141.7 ± 26.7	10.4	12.6	52.0	52.4	Moderate

Seino et al. [54]	158	42 (26.6)	2.5	24 weeks	308.11 ± 70.19	295.02 ± 68.4	8.14 ± 0.91	8.17 ± 0.80	160.8 ± 28.7	161.9 ± 31.0	6.5	6.1	—	—	Moderate

Seino et al. [54]	236	66 (32.2)	0.5 2.5 5	12 weeks	314.65 ± 64.83 302.75 ± 88.63 302.16 ± 76.73	317.62 ± 77.32	8.16 ± 0.938.07 ± 0.908.16 ± 0.96	7.88 ± 0.72	158.7 ± 28.8158.1 ± 30.3159.9 ± 34.7	153.1 ± 24.8	4.90 6.15 5.77	7.30	—	—	Moderate

Seino et al. [55]	280	82 (29.3)	1 2.5 5 10	12 weeks	320.60 ± 74.94 306.92 ± 76.13 296.81 ± 67.21 289.67 ± 70.19	311.68 ± 60.07	7.77 ± 0.798.05 ± 0.757.86 ± 0.697.95 ± 0.67	7.92 ± 0.84	152.0 ± 28.4156.1 ± 28.5149.3 ± 23.1155.3 ± 28.2	158.2 ± 33.3	4.7 4.6 4.5 6.2	5.1	—	—	Moderate

*Ertugliflozin*															

Budoff et al.∗ [20]	307	83 (25.2)	5 15	18 weeks	323.57 ± 83.87 328.33 ± 79.7	323.57 ± 81.49	8.4 ± 1.0 8.3 ± 1.0	8.3 ± 1.0	183.5 ± 49.6 174.0 ± 52.8	177.3 ± 45.6	11.6 11.1	11.6	84.8 80.2	85.5	Moderate

*Licogliflozin*															

Yokote et al. [69]	123	48 (38.1)	2.5 10 25 50	12 weeks	—	—	6.9 ± 1.0 6.7 ± 0.8 6.6 ± 0.7 6.6 ± 0.8	6.5 ± 0.6	133.33 ± 30.63 127.92 ± 23.42 126.12 ± 25.22 126.12 ± 23.42	120.72 ± 19.81	—	—	104.0 101.7 103.2 105.4	100.4	Moderate

*Sotagliflozin*															

Van Raalte et al.∗ [63]	1434	779 (49.5)	200 400	24 weeks	269.44 264.69	268.25	7.7 ± 0.8 7.6 ± 0.8	7.7 ± 0.8	—	—	21.6 21.5	21.2	—	—	Moderate

All studies were randomized-parallel-group and double-blind. ∗Multicenter studies. †Baseline. Abbreviations: I = intervention; P = placebo; eGFR = estimated glomerular filtration rate; SUA = serum uric acid; FPG = fasting plasma glucose; HbA1c = glycated hemoglobin.

**Table 2:** The results of meta-analysis for changes in SUA, HbA1c, and FPG derived from SGLTi treatment in patients with T2DM.

SGLT2i	Dose	Mean diff. [95% CI] SUA (*μ*mol/L)	*P*-value	I^2^	SGLT2i sample size	Placebo sample size	Treatment duration (week)†	Mean diff. [95% CI] HbA1c (%)	*I* ^2^	Mean diff. [95% CI] FPG (mg/dl)	*I* ^2^	Mean diff. [95% CI] body weight (kg)	*I* ^2^
Canagliflozin	Total	-36.277 [-41.621, -30.933]	0.001>	66.5%	7976	5318	24.8	-0.648	81.6%	-26.737 [-29.888, -23.586]	68.3%	-1.990 [-2.284, -1.697]	78.7%
	100	-34.479 [-42.560, -26.398]	0.001>	56.4%	1031	953	22.8	[-0.735, -0.561]	73.2%	-25.175 [-30.262, -20.087]	71.2%	-1.769 [-2.052, -1.485]	56.7%
	300	-40.692 [-47.151, -34.232]	0.001>	24.8%	957	893	22.3	-0.594	85.4%	-28.874 [-34.204, -23.545]	71.4%	-2.286 [-2.777, -1.796]	84.1%

Dapagliflozin	Total	-35.176 [-39.687, -30.665]	0.001>	73.9%	4800	2519	24.2	[-0.711, -0.477]	93.7%	-19.450 [-21.797, -17.102]	96.7%	-1.510 [-1.706, -1.336]	98.3%
	2.5	-31.592 [-41.172, -22.012]	0.001>	66.0%	825	733	23.0	-0.719	94.5%	-15.368 [-21.375, -9.36]	97.9%	-1.271 [-1.488, -1.053]	80.2%
	5	-33.595 [-41.071, -26.118]	0.001>	69.0%	1340	1269	27.7	[-0.873, -0.564]	89.1%	-19.675 [-23.737, -15.612]	94.6%	-1.405 [-1.762, -1.048]	99.0%
	10	-35.284 [-42.538, -28.029]	0.001>	78.7%	2327	2196	23.5	-0.490	91.1%	-20.734 [-24.225, -17.243]	93.4%	-1.700 [-1.935, -1.464]	97.5%

Empagliflozin	Total	-40.980 [-47.632, -34.328]	0.001>	84.9%	9039	4242	28.0	[-0.536, -0.444]	96.1%	-25.911 [-29.100, -22.721]	80.6%	-1.765 [-2.038, -1.493]	99.7%
	10	-40.485 [-50.361, -30.609]	0.001>	%84.5	4499	3978	27.89	-0.485	92.0%	-25.265 [-30.262, -20.267]	78.5%	-1.836 [-2.042, -1.630]	98.2%
	25	-38.791 [-49.643, -27.939]	0.001>	85.8%	4492	4154	29.08	[-0.588, -0.381]	97.7%	-25.345 [-30.581, -20.110]	83.3%	-1.678 [-2.158, -1.199]	99.8%

Ipragliflozin	Total	-18.850 [-27.202, -10.499]	0.001>	59.0%	583	181	12.4	-0.470	91.4%	-31.593 [-42.446, -20.740]	93.0%	-1.365 [-1.613, -1.098]	99.3%

Tofogliflozin	Total	-19.476 [-27.402, -11.550]	0.001>	0.0%	299	114	20.3	[-0.543, -0.396]	**—**	**—**	**—**	-2.069 [-2.676, -1.461]	79.1%

Luseogliflozin	Total	-24.269 [-33.316, -15.223]	0.001>	66.3%	579	240	15.6	-0.490	71.5%	-22.566 [-27.709, -17.243]	73.9%	-1.528 [-1.909, -1.147]	76.8%

All∗	Total	-34.076 [-37.006, -31.146]	0.001>	78.8%	23494	12721	**—**	[-0.564, -0.416]	**—**	—	—	—	—

Abbreviations: SUA = serum uric acid; FPG = fasting plasma glucose; HbA1c = glycated hemoglobin; CI = confidence interval; Diff = difference.

**Table 3:** The results of metaregression analysis on the effects of SGLT2i on SUA reduction based on duration of diabetes, treatment duration, and SGLT2i dosage.

	Canagliflozin			Dapagliflozin				Empagliflozin		
	100 mg	300 mg	Total	2.5 mg	5 mg	10 mg	Total	10 mg	25 mg	Total
*Week*										
Coefficient	0.329 [-1.400, 2.059]	-0.524 [-1.674, 0.625]	0.026 [-0.751, 0.805]	—	-0.030 [-0.587, 0.523]	0.236 [-0.302, 0.776]	0.124 [-0.255, 0.505]	0.550 [0.068, 1.03]	0.634 [0.089, 1.179]	0.607 [0.282, 0.931]
*P*-value	0.709	0.371	0.946	—	0.913	0.389	0.520	0.025	0.022	<0.001
*Dosage*										
Coefficient	—	—	-0.013 [-0.046, 0.018]	—	—	—	-0.606 [-2.055, 0.842]	—	—	-0.278 [-0.788, 0.232]
*P*-value	—	—	0.411	**—**	—	—	0.411	—	—	0.285
*Duration of diabetes*										
Coefficient	2.061 [-0.359, 4.481]	1.286 [-0.691, 3.264]	1.581 [0.148, 3.014]	1.746 [0.503, 2.990]	2.109 [1.254, 2.964]	2.076 [0.889, 3.263]	1.906 [1.218, 2.594]	—	—	—
*P*-value	0.095	0.202	0.03	0.005	<0.001	<0.001	<0.001	—	—	—

## Data Availability

There is no raw data associated with this article.

## Supplementary Materials

Supplementary Materials Table S1: search strategy. Figure S1: meta-analysis of all canagliflozin studies to determine the drug efficacy in serum uric acid reduction. Figure S2: meta-analysis of all dapagliflozin studies to determine the drug efficacy in serum uric acid reduction. Figure S3: meta-analysis of all empagliflozin studies to determine the drug efficacy in serum uric acid reduction. Figure S4: scatterplots of metaregression on canagliflozin variables (weeks of treatment, drug dosage, and duration of diabetes). Figure S5: scatterplots of metaregression on dapagliflozin variables (weeks of treatment, drug dosage, and duration of diabetes). Figure S6: scatterplots of metaregression on empagliflozin variables (weeks of treatment, drug dosage, and duration of diabetes). Figure S7: meta-analysis of all canagliflozin studies to determine the drug effects on HbA1c. Figure S8: meta-analysis of all dapagliflozin studies to determine the drug effects on HbA1c. Figure S9: meta-analysis of all empagliflozin studies to determine the drug effects on HbA1c. Figure S10: meta-analysis of all canagliflozin studies to determine the drug effects on FPG. Figure S11: meta-analysis of all dapagliflozin studies to determine the drug effects on FPG. Figure S12: meta-analysis of all empagliflozin studies to determine the drug effects on FPG. Figure S13: meta-analysis of all canagliflozin studies to determine the drug effects on body weight. Figure S14: meta-analysis of all dapagliflozin studies to determine the drug effects on body weight. Figure S15: meta-analysis of all empagliflozin studies to determine the drug effects on body weight.
