# Supplementary material for: Impacts of Sodium/Glucose Cotransporter-2 Inhibitors on Circulating Uric Acid Concentrations: A Systematic Review and Meta-Analysis
Source: J Diabetes Res. 2022 Feb 17;2022:7520632. doi: 10.1155/2022/7520632 (PMC8872662; doi:10.1155/2022/7520632)

| **Table S1: Search strategy** | |
| --- | --- |
| **Pubmed** | |
| **Query** | **Search** |
| ("sodium-glucose cotransporter 2 inhibitors"[title/abstract] OR " sodium-glucose transporter 2 inhibitors"[title/abstract] OR SGLT2i[title/abstract] OR "SGLT 2i"[title/abstract] OR SGLT2Is[title/abstract] OR "SGLT 2is"[title/abstract] OR atigliflozin [title/abstract] OR AVE2268 [title/abstract] OR Dapaglifozin [title/abstract] OR PF04971729[title/abstract] OR "BMS 512148"[title/abstract] OR bexagliflozin [title/abstract] OR egt0001442 [title/abstract] OR egt0001474 [title/abstract] OR canagliflozin[title/abstract] OR invokana[title/abstract] OR " JNJ 28431754"[title/abstract] OR dapagliflozin[title/abstract] OR farxiga[title/abstract] OR egt0001474[title/abstract] OR empagliflozin[title/abstract] OR jardiance[title/abstract] OR "BI 10773"[title/abstract] OR BI10773 [title/abstract] OR ertugliflozin[title/abstract] OR ipragliflozin[title/abstract] OR suglat[title/abstract] OR asp1941[title/abstract] OR isis-sglt2rx[title/abstract] OR LX4211[title/abstract] OR luseogliflozin[title/abstract] OR lusefi[title/abstract] OR TS071[title/abstract] OR remogliflozin[title/abstract] OR bhv091009[title/abstract] OR sergliflozin[title/abstract] OR shr3824[title/abstract] OR sotagliflozin[title/abstract] OR "sodium-glucose cotransporter 2 inhibitor"[title/abstract] OR tofogliflozin[title/abstract] OR apleway[title/abstract] OR deberza[title/abstract] OR CSG452[title/abstract] OR (((((Na+/glucose[title/abstract] OR "sodium glucose"[title/abstract] OR "sodium dependent glucose"[title/abstract]) AND (transporter[title/abstract] OR cotransporter[title/abstract] OR "co transporter"[title/abstract])) OR ((SGLT2[title/abstract] OR SGLT 2[title/abstract]))) AND (inhibitor*[title/abstract))) | **#1** (SGLT2I) |
| (Hyperuric*[title/abstract] OR Hyporuric*[title/abstract] OR "uric-acid"[title/abstract] OR "acid-uric"[title/abstract] OR urat*[title/abstract] OR uric[title/abstract] OR "Sodium Acid"[title/abstract] OR 2,6,8-Trihydroxypurine[title/abstract] OR Trihydroxypurine[title/abstract] OR Trioxopurine[title/abstract] OR "Sodium Monohydrate"[title/abstract] OR urate[title/abstract] OR uric acid[title/abstract] OR BUN[title/abstract] OR Creatinine[title/abstract] OR "cardiometabolic biomarker*"[title/abstract] OR "cardiometabolic parameter*"[title/abstract] OR "Cardiometabolic Biological Marker*"[title/abstract] OR "cardiometabolic profile*"[title/abstract] OR "cardiometabolic risk*"[title/abstract] OR "Cardiometabolic Biologic Marker*"[title/abstract] OR "Cardiometabolic Serum Marker*"[title/abstract] OR "Cardiometabolic Laboratory Marker*"[title/abstract] OR "Cardiometabolic outcome*"[title/abstract])) | **#2** (Uric acid) |
| **#1 AND #2** | **Final** |
| **Scopus** | |
| TITLE-ABS-KEY("sodium-glucose cotransporter 2 inhibitors" OR "sodium-glucose transporter 2 inhibitors" OR SGLT2i OR "SGLT 2i" OR SGLT2Is OR "SGLT 2is" OR atigliflozin or AVE2268 OR Dapaglifozin or PF04971729 or "BMS 512148" OR bexagliflozin OR egt0001442 OR egt0001474 OR canagliflozin OR invokana or "JNJ 28431754" OR dapagliflozin OR farxiga OR egt0001474 OR empagliflozin OR jardiance or "BI 10773" OR BI10773 OR ertugliflozin OR ipragliflozin OR suglat OR asp1941 OR isis-sglt2rx OR LX4211 OR luseogliflozin OR "lusefior TS071" OR "sodium-glucose cotransporter 2 inhibitor" or remogliflozin OR bhv091009 OR sergliflozin OR shr3824 OR sotagliflozin OR tofogliflozin OR apleway OR deberzaor CSG452 OR ( ( na+/glucose OR "sodium glucose" OR "sodium dependent glucose" ) AND ( transporter OR cotransporter OR "co transporter" ) ) OR ((SGLT2 OR SGLT 2) AND inhibitor*)) | **#1** (SGLT2I) |
| TITLE-ABS-KEY( Hyperuric* or Hyporuric* or uric-acid or "acid-uric" or urat* or uric or "Sodium Acid" or 2,6,8-Trihydroxypurine or Trihydroxypurine or Trioxopurine or Ammonium or "Sodium Monohydrate" or BUN or Creatinine or "cardiometabolic biomarker*" or "cardiometabolic parameter*" or "cardiometabolic parameter*" or "Cardiometabolic Biological Marker*" or "cardiometabolic profile*" or urate or "uric acid" or "cardiometabolic risk*" or "Cardiometabolic Biologic Marker*" or "Cardiometabolic Serum Marker*" or "Cardiometabolic Laboratory Marker*" or "Cardiometabolic outcome*") | **#2** (Uric acid) |
| **#1 AND #2** | **Final** |
| **Embase** | |
| (('sodium-glucose cotransporter 2 inhibitors':ab,ti OR 'sodium-glucose transporter 2 inhibitors':ab,ti OR sglt2i:ab,ti OR 'sglt 2i':ab,ti OR sglt2is:ab,ti OR 'sglt 2is':ab,ti OR atigliflozin:ab,ti OR ave2268:ab,ti OR dapaglifozin:ab,ti OR pf04971729:ab,ti OR 'bms 512148':ab,ti OR bexagliflozin:ab,ti OR egt0001442:ab,ti OR canagliflozin:ab,ti OR invokana:ab,ti OR 'jnj 28431754':ab,ti OR dapagliflozin:ab,ti OR farxiga:ab,ti OR egt0001474:ab,ti OR empagliflozin:ab,ti OR jardiance:ab,ti OR 'bi 10773':ab,ti OR bi10773:ab,ti OR ertugliflozin:ab,ti OR ipragliflozin:ab,ti OR suglat:ab,ti OR asp1941:ab,ti OR 'isis sglt2rx':ab,ti OR lx4211:ab,ti OR luseogliflozin:ab,ti OR 'lusefior ts071':ab,ti OR remogliflozin:ab,ti OR bhv091009:ab,ti OR sergliflozin:ab,ti OR 'sodium-glucose cotransporter 2 inhibitor':ti,ab or shr3824:ab,ti OR sotagliflozin:ab,ti OR tofogliflozin:ab,ti OR apleway:ab,ti OR 'deberzaor csg452':ab,ti OR (('na+/glucose':ab,ti OR 'sodium glucose':ab,ti OR 'sodium dependent glucose':ab,ti) AND (transporter:ab,ti OR cotransporter:ab,ti OR 'co transporter':ab,ti)) OR ((sglt2:ab,ti OR 'sglt 2':ab,ti) AND inhibitor*:ab,ti)) | **#1** (SGLT2I) |
| (hyperuric*:ti,ab OR hyporuric*:ti,ab OR 'uric acid':ti,ab OR 'acid-uric':ti,ab OR urat*:ti,ab OR uric:ti,ab OR 'sodium acid':ti,ab OR '2,6,8 trihydroxypurine':ti,ab OR trihydroxypurine:ti,ab OR trioxopurine:ti,ab OR ammonium:ti,ab OR 'sodium monohydrate':ti,ab OR bun:ti,ab OR creatinine:ti,ab or urate:ti,ab or 'uric acid':ti,ab OR 'cardiometabolic biomarker*':ti,ab OR 'cardiometabolic parameter*':ti,ab OR 'cardiometabolic biological marker*':ti,ab OR 'cardiometabolic profile*':ti,ab OR 'cardiometabolic risk*':ti,ab OR 'cardiometabolic biologic marker*':ti,ab OR 'cardiometabolic serum marker*':ti,ab OR 'cardiometabolic laboratory marker*':ti,ab OR 'cardiometabolic outcome*':ti,ab)) | **#2** (Uric acid) |
| controlled study OR major clinical study OR randomized controlled trial OR clinical trial OR double blind procedure OR randomized controlled trial topic OR multicenter study OR phase 3 clinical trial OR comparative effectiveness OR clinical trial topic OR phase 3 clinical trial topic OR  controlled clinical trial OR crossover procedure OR clinical study OR parallel design OR phase 2 clinical trial OR comparative study | **Filters (limited to)** |
| **#1 AND #2** | **Final** |
| **Web Of Science** | |
| ((TI=("sodium-glucose cotransporter 2 inhibitors" OR "sodium-glucose transporter 2 inhibitors" OR SGLT2i OR "SGLT 2i" OR SGLT2Is OR "SGLT 2is" OR atigliflozin or AVE2268 OR Dapaglifozin or PF04971729 or "BMS 512148" OR bexagliflozin OR egt0001442 OR egt0001474 OR canagliflozin OR invokana or "JNJ 28431754" OR dapagliflozin OR farxiga OR egt0001474 OR empagliflozin OR jardiance or "BI 10773" OR BI10773 OR "sodium-glucose cotransporter 2 inhibitor" or ertugliflozin OR ipragliflozin OR suglat OR asp1941 OR isis-sglt2rx OR LX4211 OR luseogliflozin OR "lusefior TS071" OR remogliflozin OR bhv091009 OR sergliflozin OR shr3824 OR sotagliflozin OR tofogliflozin OR apleway OR deberzaor CSG452 OR ( ( na+/glucose OR "sodium glucose" OR "sodium dependent glucose" ) AND ( transporter OR cotransporter OR "co transporter" ) ) OR ((SGLT2 OR SGLT 2) AND inhibitor*)) OR AB=("sodium-glucose cotransporter 2 inhibitors" OR "sodium-glucose transporter 2 inhibitors" OR SGLT2i OR "SGLT 2i" OR SGLT2Is OR "SGLT 2is" OR atigliflozin or AVE2268 OR Dapaglifozin or PF04971729 or "BMS 512148" OR bexagliflozin OR egt0001442 OR egt0001474 OR canagliflozin OR invokana or "JNJ 28431754" OR dapagliflozin OR farxiga OR egt0001474 OR empagliflozin OR jardiance or "BI 10773" OR BI10773 OR "sodium-glucose cotransporter 2 inhibitor" or ertugliflozin OR ipragliflozin OR suglat OR asp1941 OR isis-sglt2rx OR LX4211 OR luseogliflozin OR "lusefior TS071" OR remogliflozin OR bhv091009 OR sergliflozin OR shr3824 OR sotagliflozin OR tofogliflozin OR apleway OR deberzaor CSG452 OR ( ( na+/glucose OR "sodium glucose" OR "sodium dependent glucose" ) AND ( transporter OR cotransporter OR "co transporter" ) ) OR ((SGLT2 OR SGLT 2) AND inhibitor*))) | **#1** (SGLT2I) |
| (TI=( Hyperuric* or Hyporuric* or uric-acid or "acid-uric" or urat* or uric or "Sodium Acid" or 2,6,8-Trihydroxypurine or Trihydroxypurine or Trioxopurine or Ammonium or "Sodium Monohydrate" or BUN or Creatinine or "cardiometabolic biomarker*" or "cardiometabolic parameter*" or "cardiometabolic parameter*" or "Cardiometabolic Biological Marker*" or "cardiometabolic profile*" or "cardiometabolic risk*" or urate or "uric acid" or "Cardiometabolic Biologic Marker*" or "Cardiometabolic Serum Marker*" or "Cardiometabolic Laboratory Marker*" or "Cardiometabolic outcome*") OR AB=( Hyperuric* or Hyporuric* or uric-acid or "acid-uric" or urat* or uric or "Sodium Acid" or 2,6,8-Trihydroxypurine or Trihydroxypurine or Trioxopurine or Ammonium or urate or "uric acid" or "Sodium Monohydrate" or BUN or Creatinine or "cardiometabolic biomarker*" or "cardiometabolic parameter*" or "cardiometabolic parameter*" or "Cardiometabolic Biological Marker*" or "cardiometabolic profile*" or "cardiometabolic risk*" or "Cardiometabolic Biologic Marker*" or "Cardiometabolic Serum Marker*" or "Cardiometabolic Laboratory Marker*" or "Cardiometabolic outcome*"))) | **#2** (Uric acid) |
| **#1 AND #2** | **Final** |

Figure S1: Meta-analysis of all canagliflozin studies to determine the drug efficacy in serum uric acid reduction.


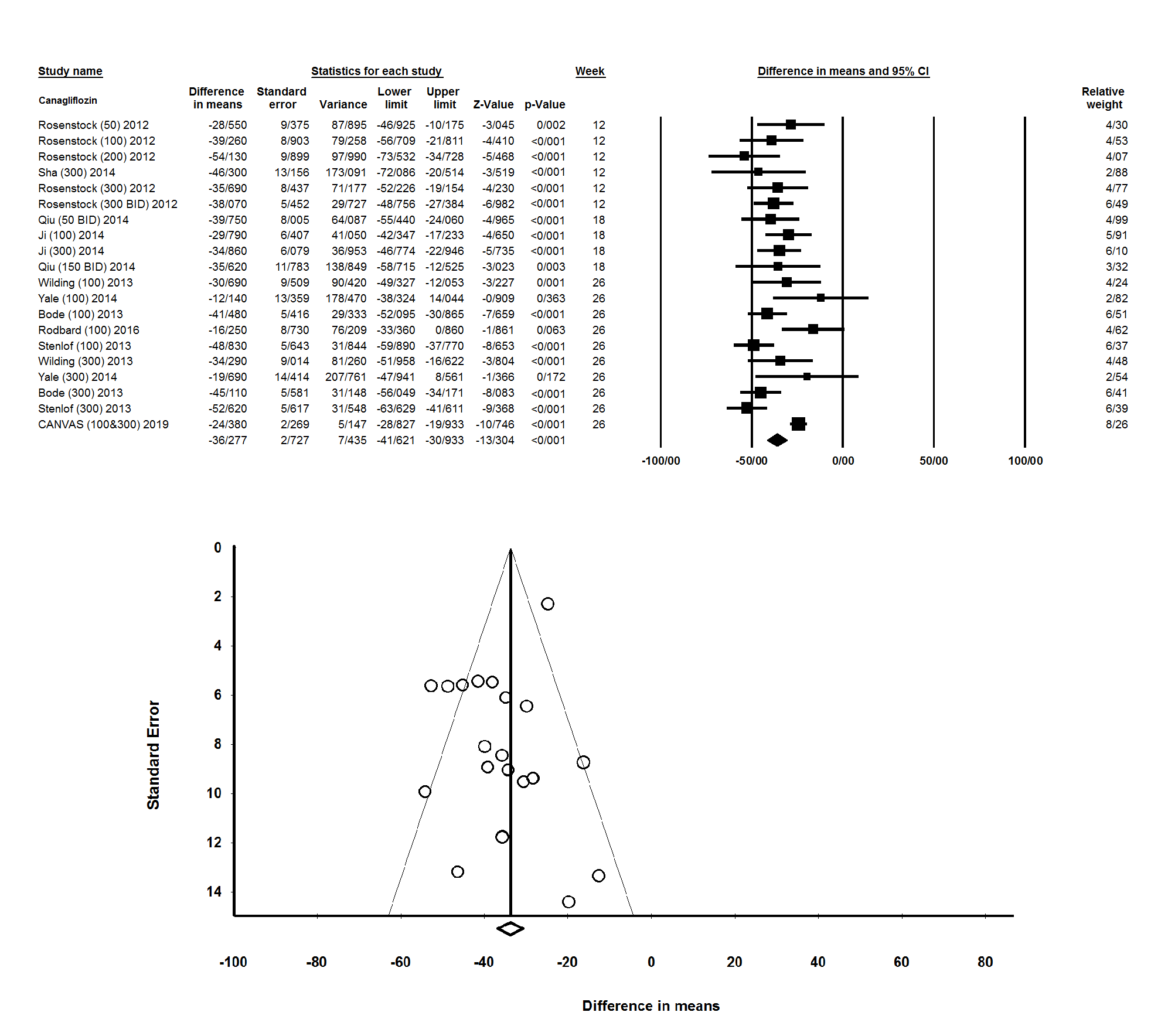


Figure S2: Meta-analysis of all dapagliflozin studies to determine the drug efficacy in serum uric acid reduction.


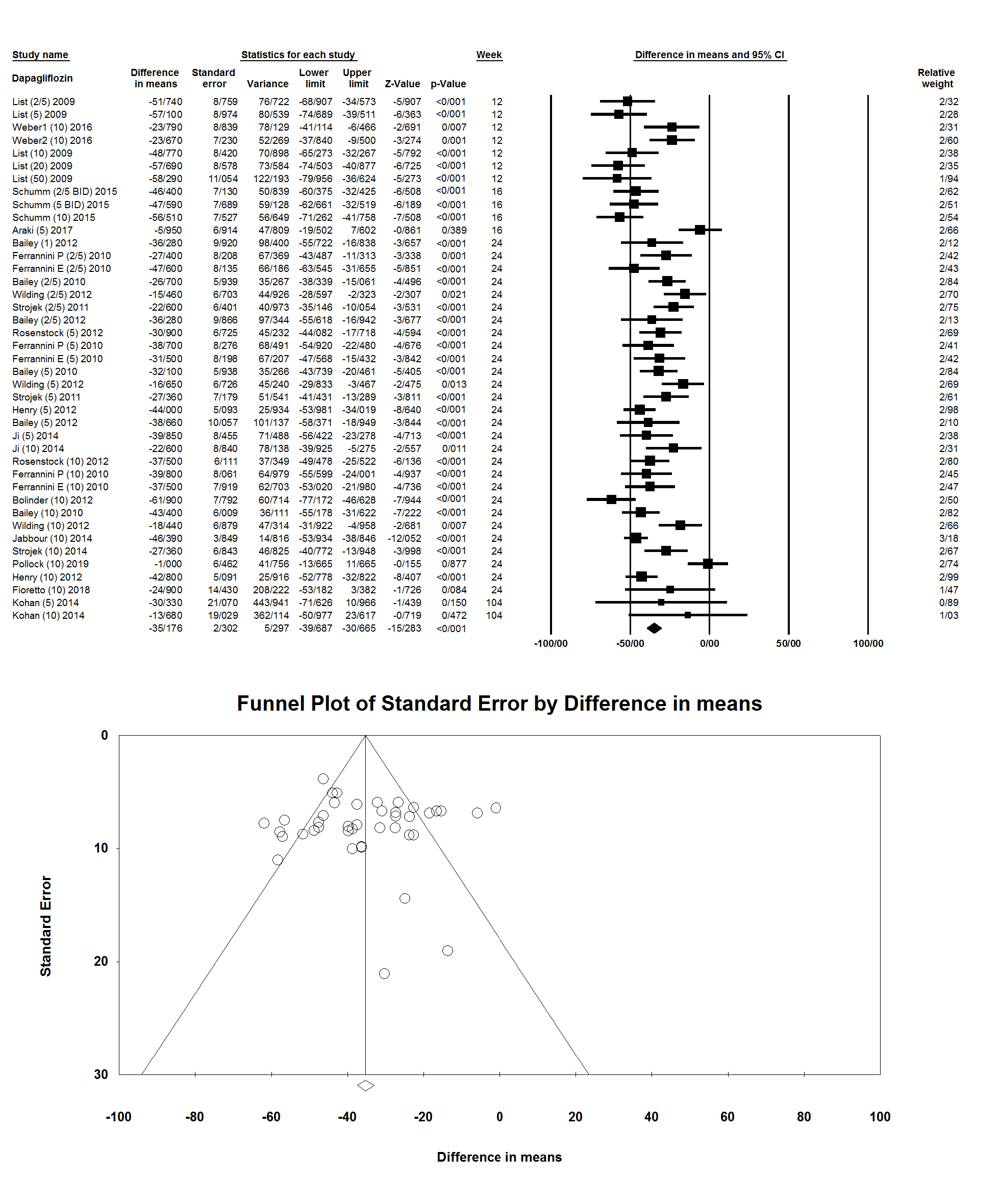


Figure S3: Meta-analysis of all empagliflozin studies to determine the drug efficacy in serum uric acid reduction.


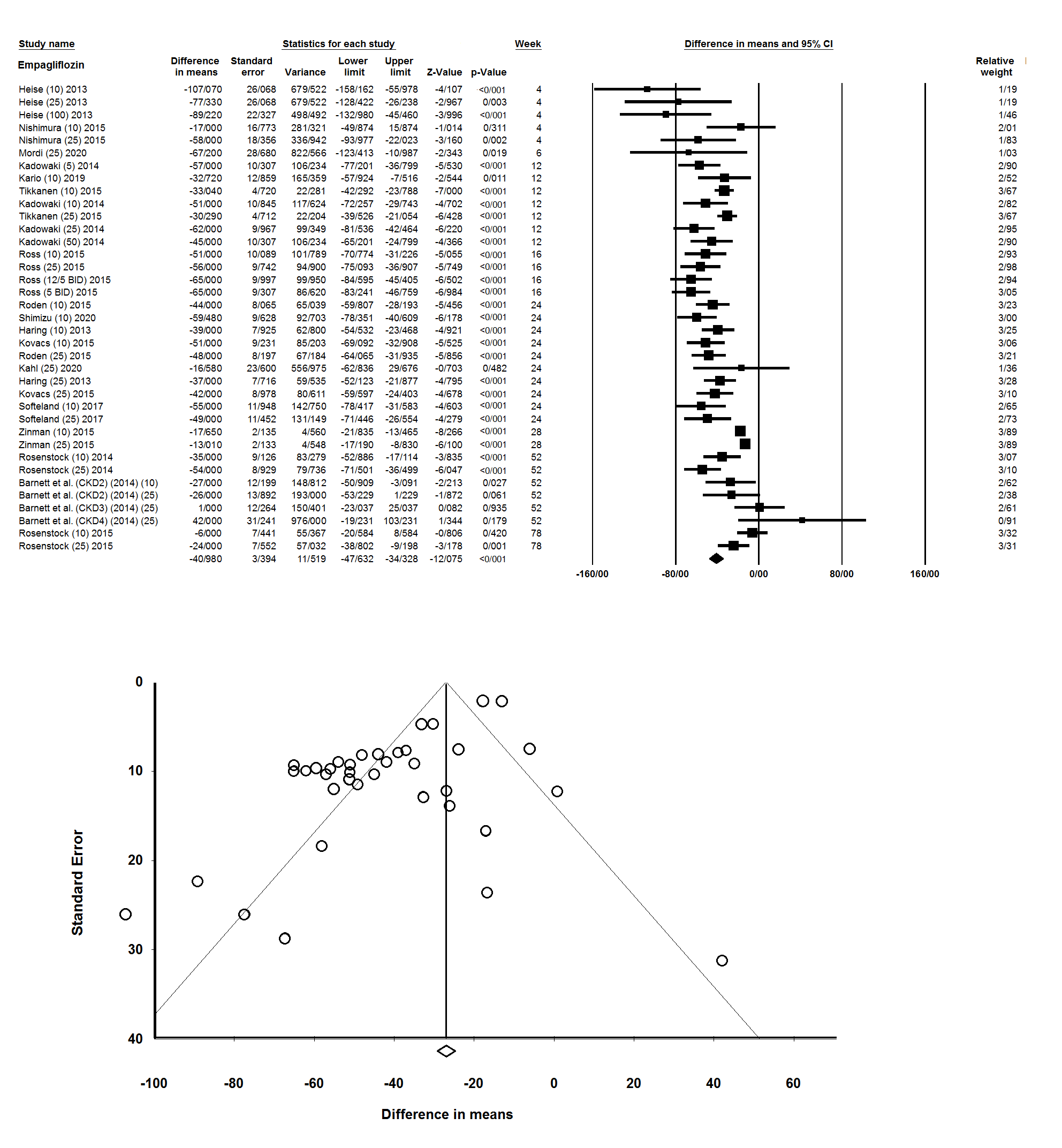


Figure S4: Scatterplots of meta-regression on canagliflozin variables (Weeks of treatment, drug dosage, and duration of diabetes)


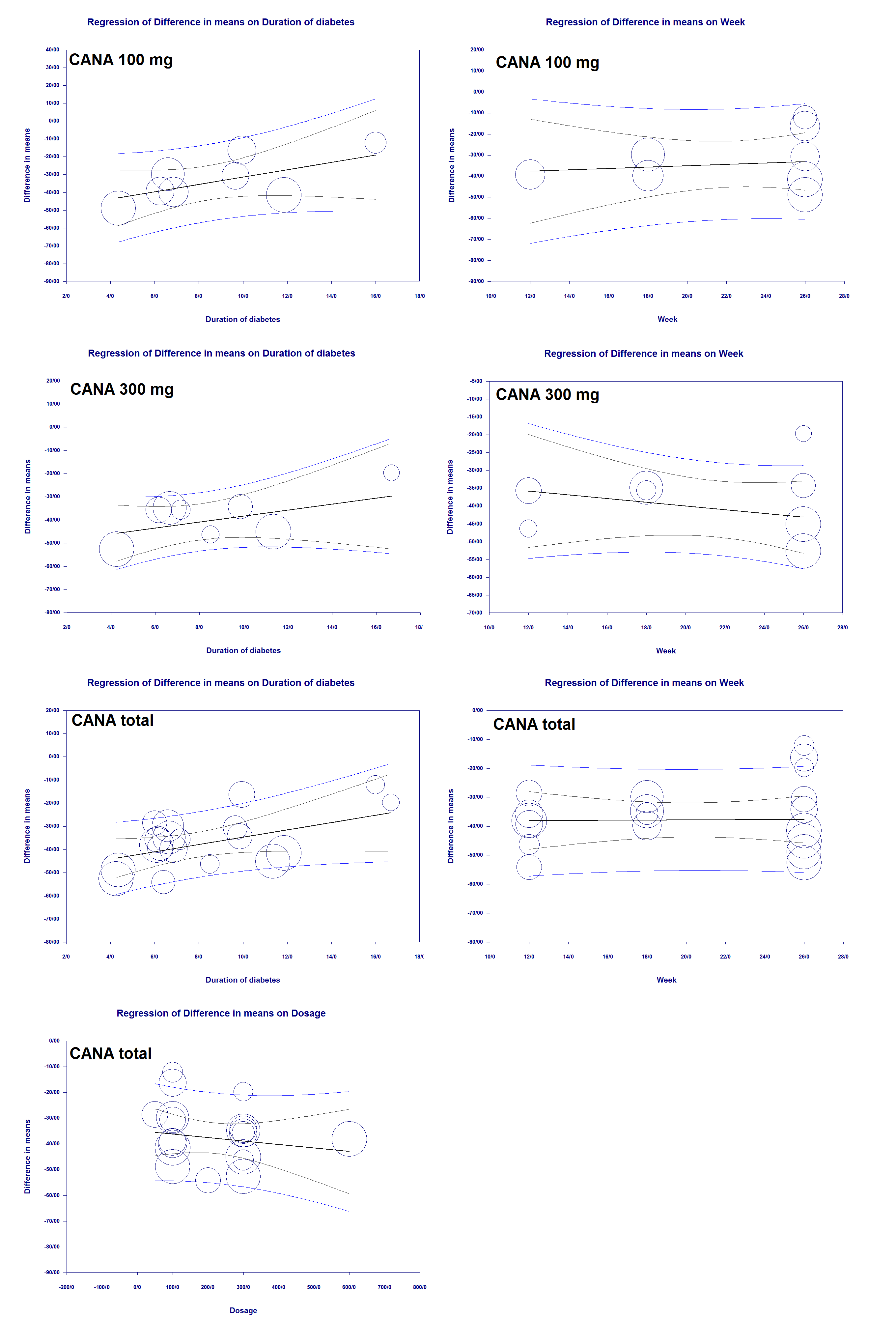


Figure S5: Scatterplots of meta-regression on dapagliflozin variables (Weeks of treatment, drug dosage, and duration of diabetes)


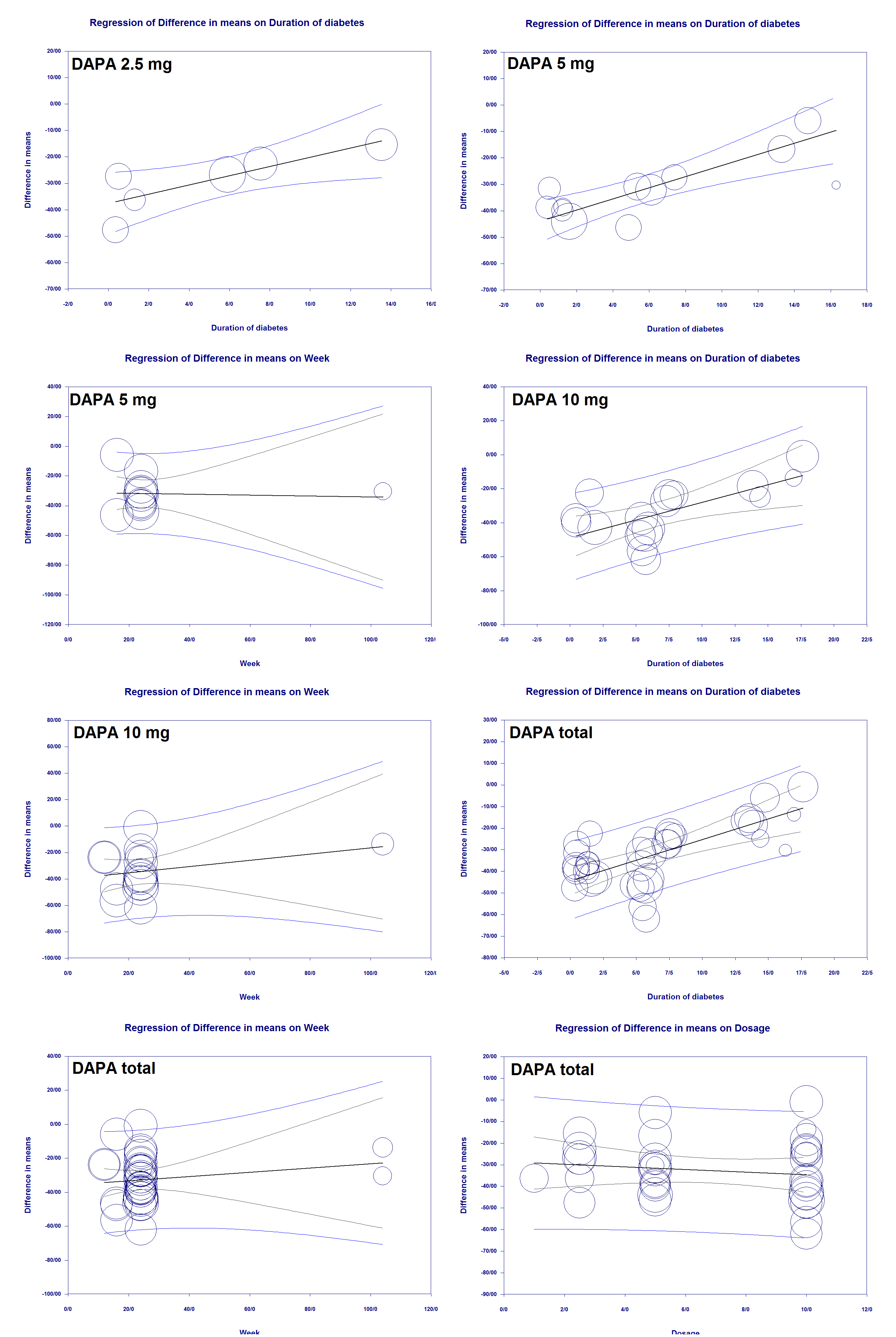


Figure S6: Scatterplots of meta-regression on empagliflozin variables (Weeks of treatment, drug dosage, and duration of diabetes)


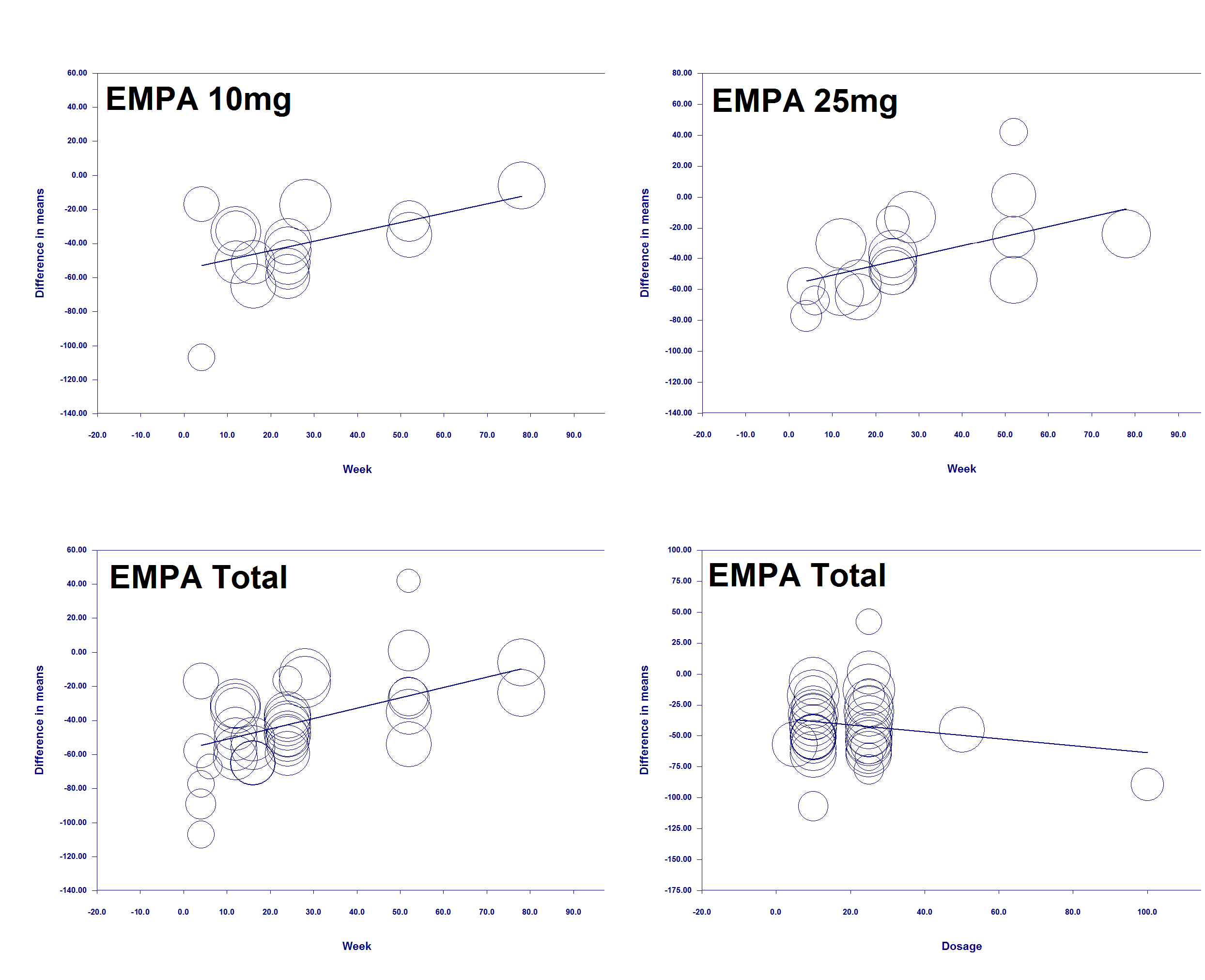


Figure S7: Meta-analysis of all canagliflozin studies to determine the drug effects on HbA1c.


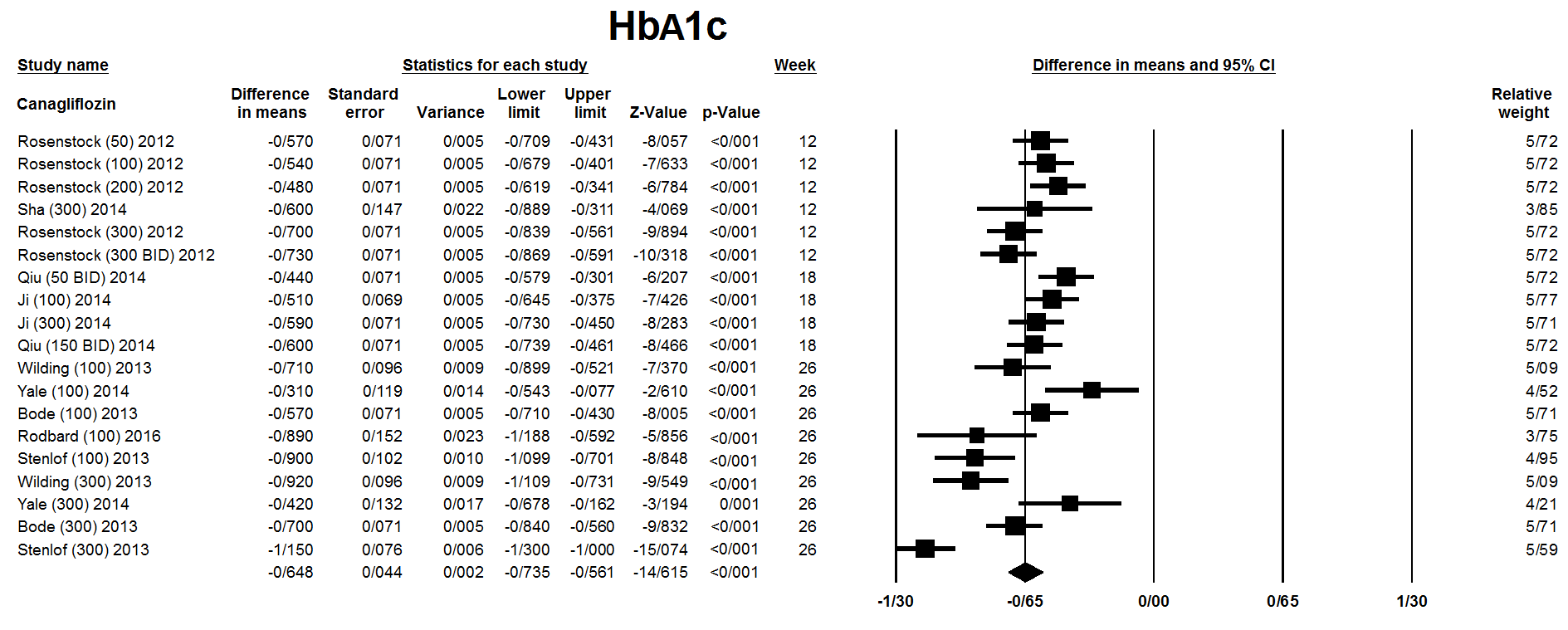


Figure S8: Meta-analysis of all dapagliflozin studies to determine the drug effects on HbA1c.


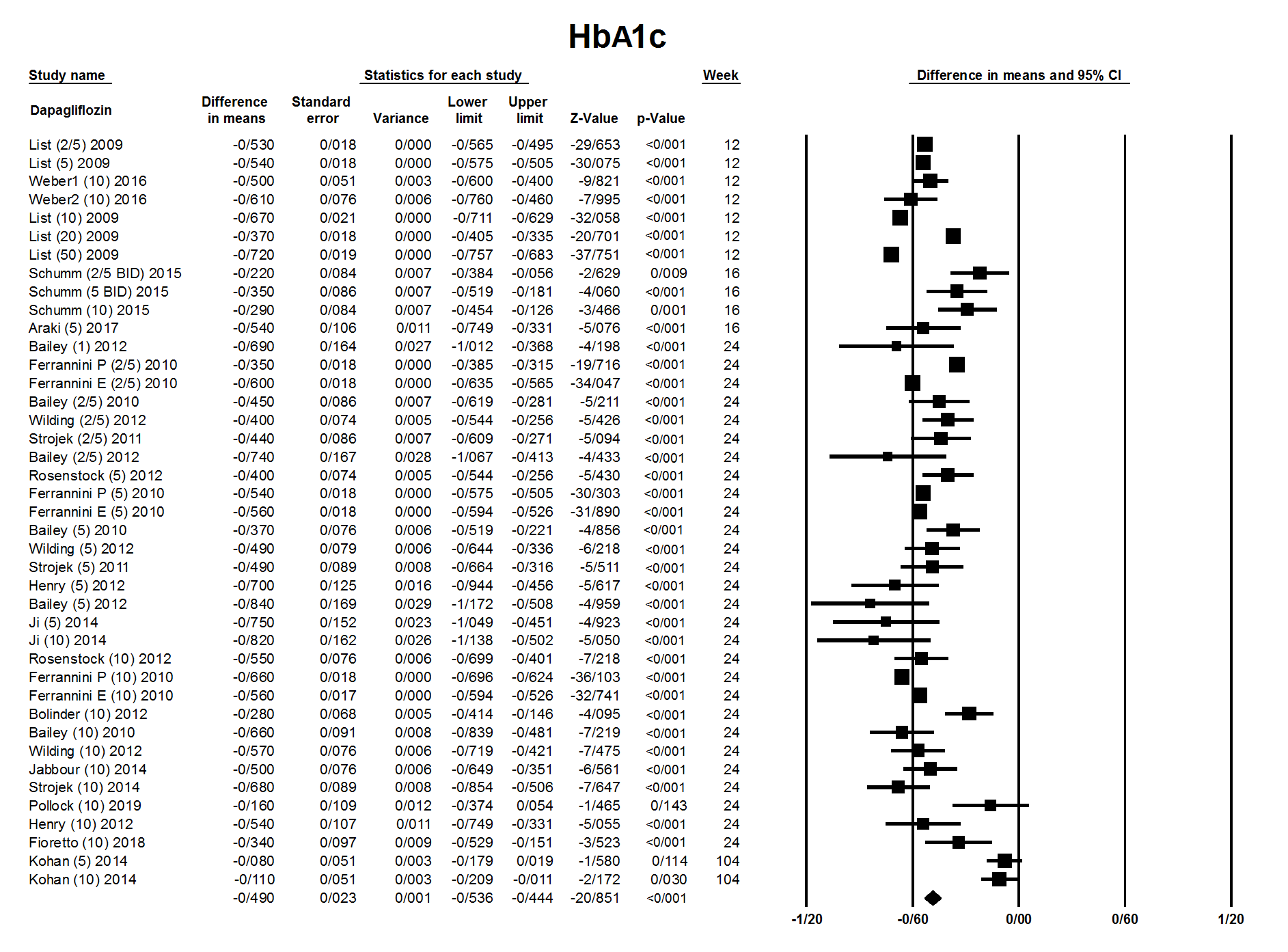


Figure S9: Meta-analysis of all empagliflozin studies to determine the drug effects on HbA1c.


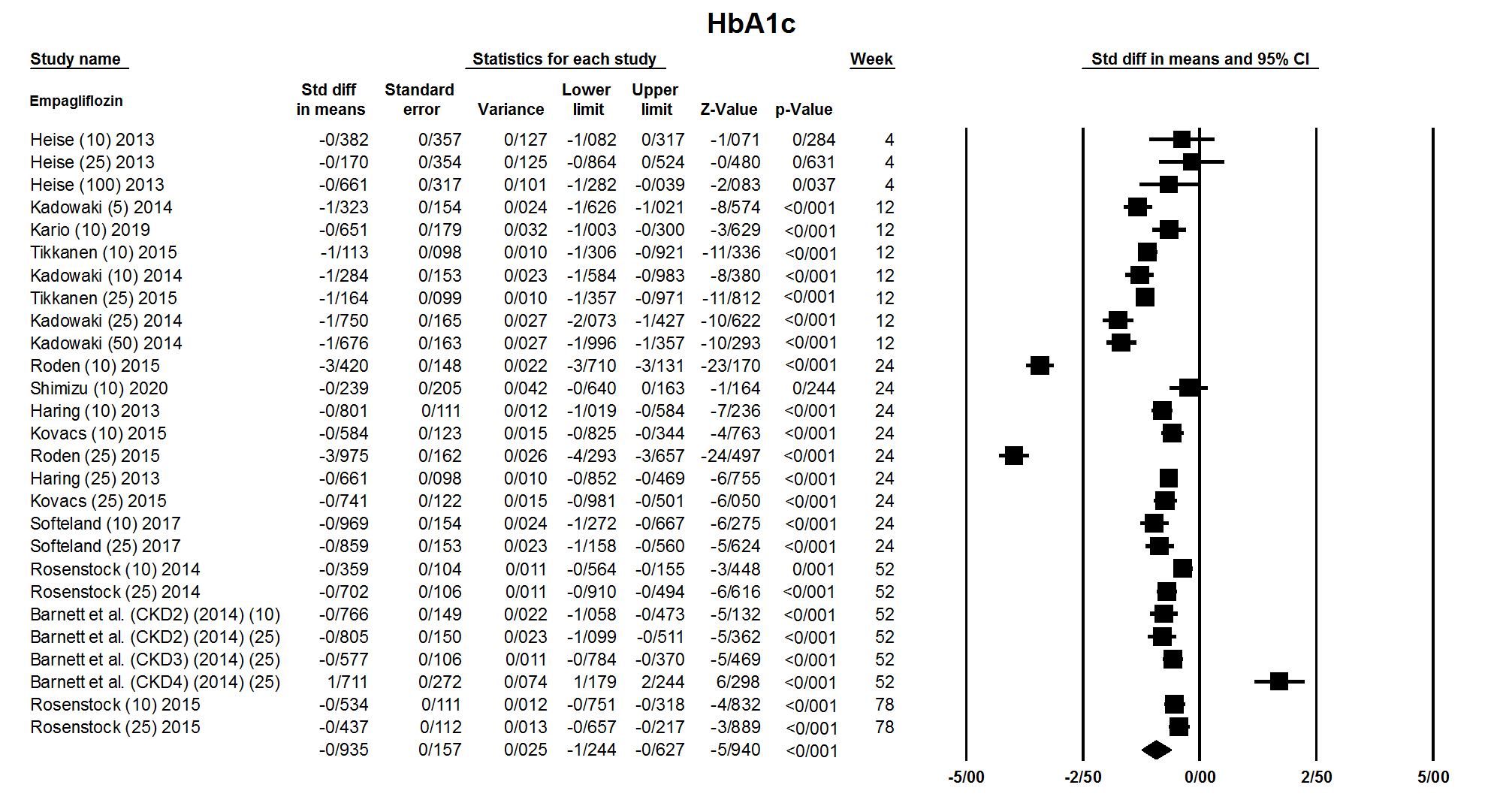


Figure S10: Meta-analysis of all canagliflozin studies to determine the drug effects on FPG.


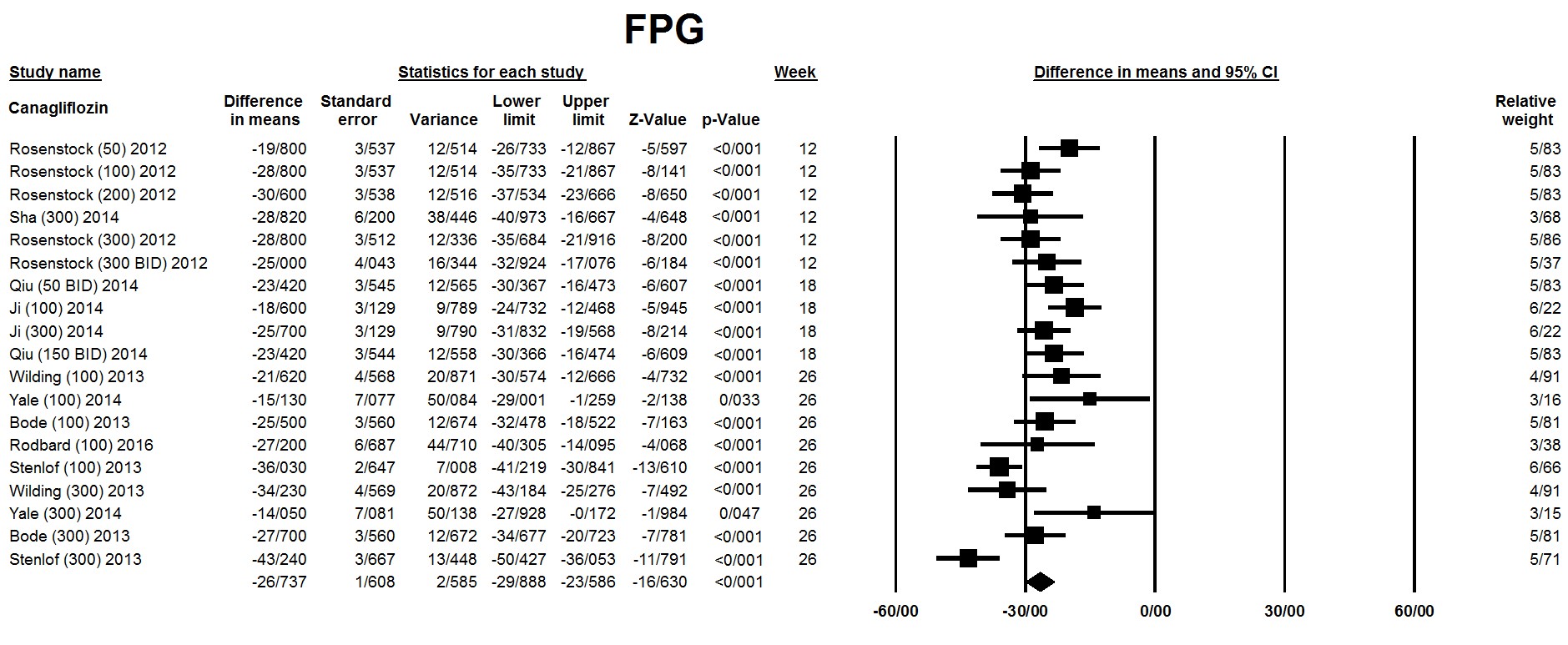


Figure S11: Meta-analysis of all dapagliflozin studies to determine the drug effects on FPG.


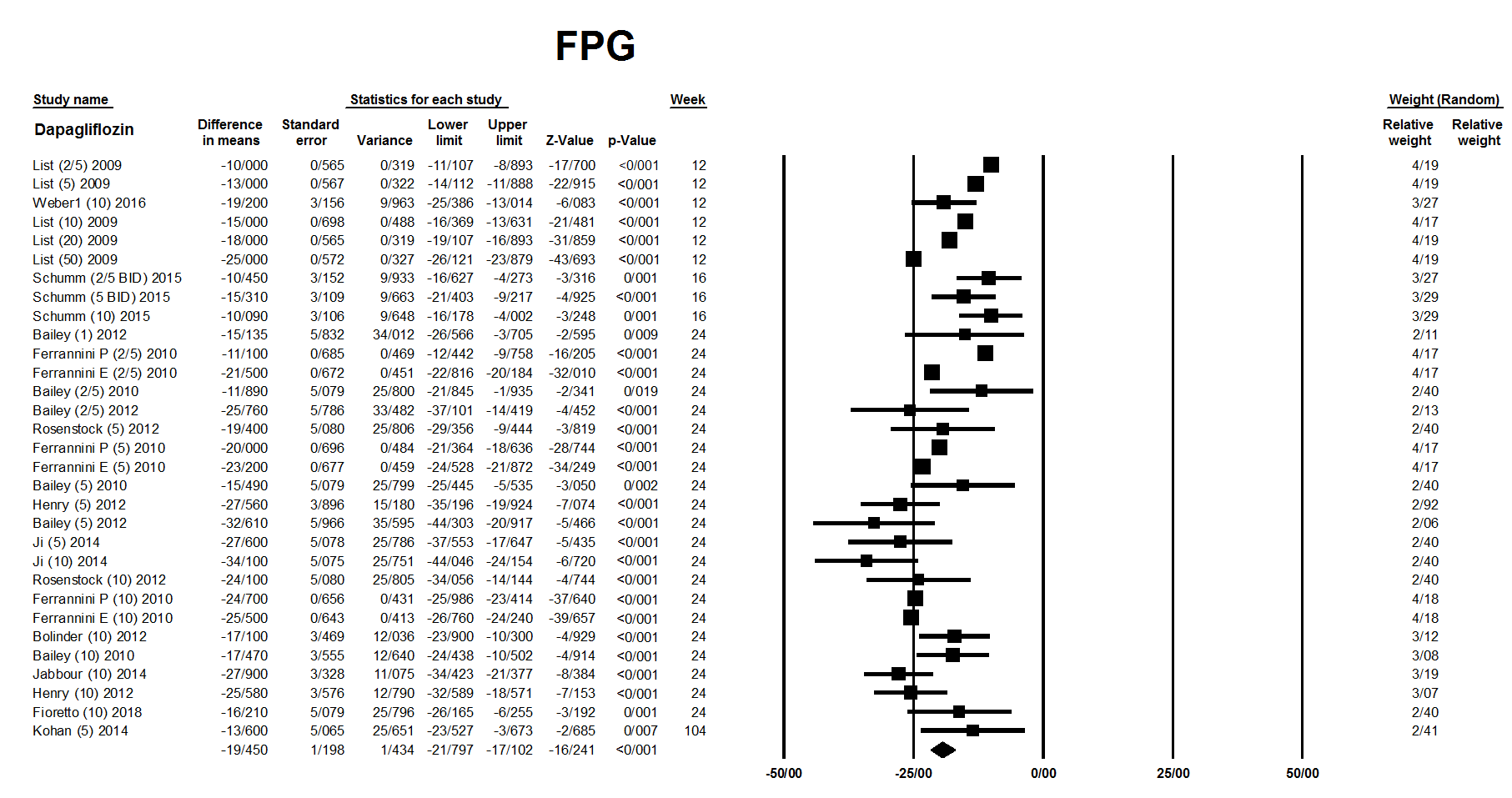


Figure S12: Meta-analysis of all empagliflozin studies to determine the drug effects on FPG.


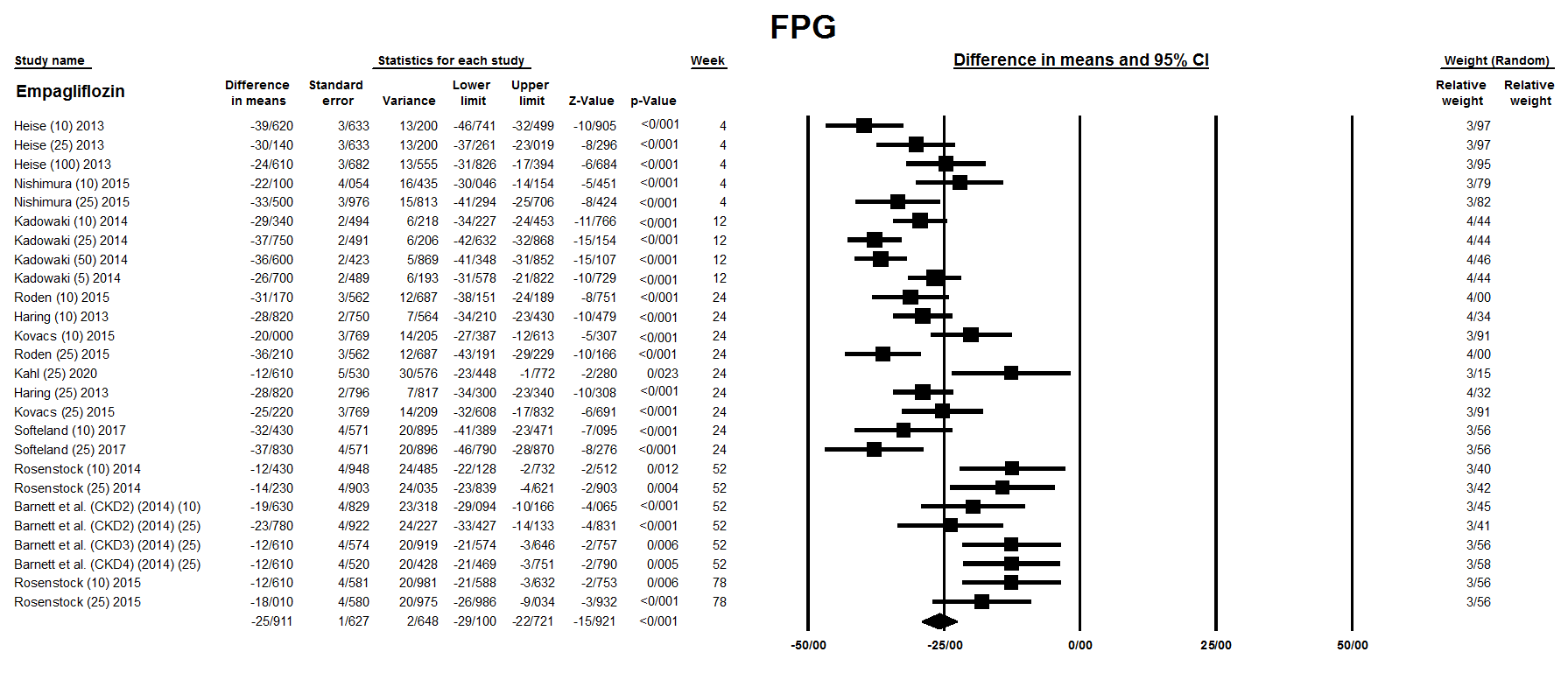


Figure S13: Meta-analysis of all canagliflozin studies to determine the drug effects on body weight.


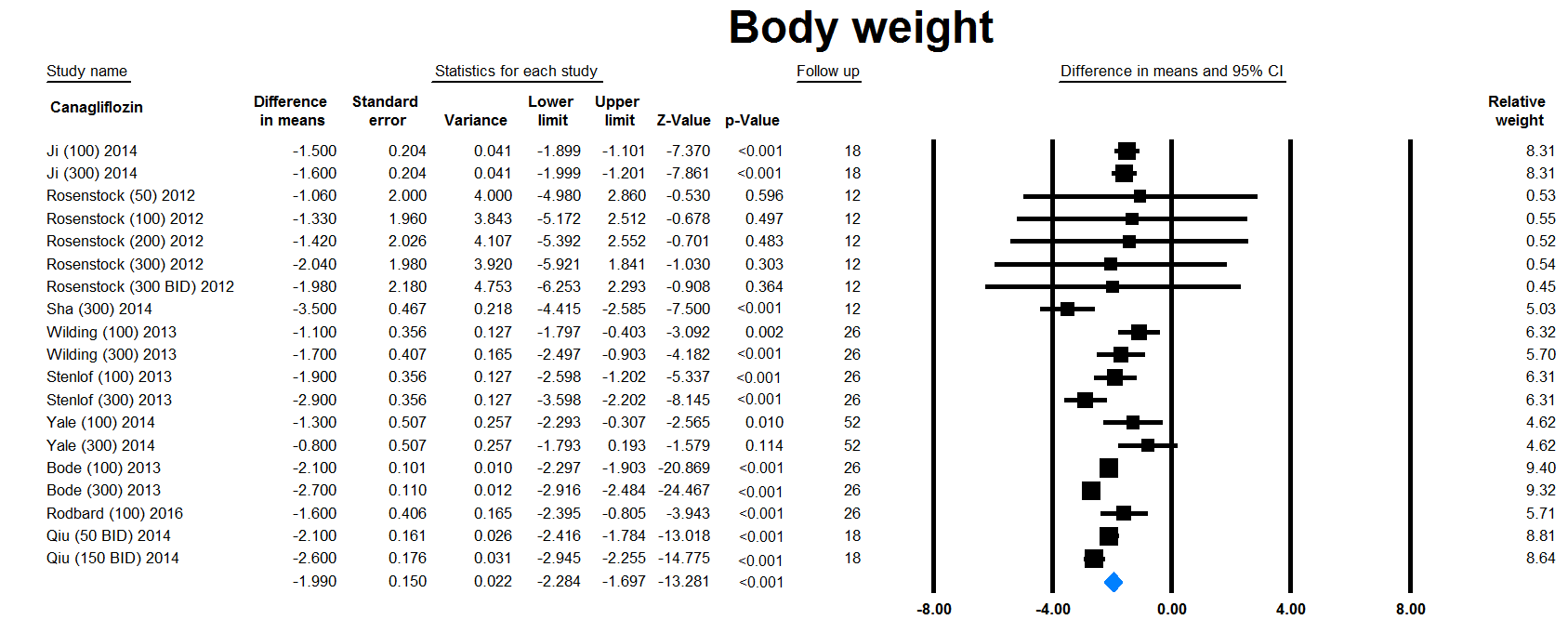


Figure S14: Meta-analysis of all dapagliflozin studies to determine the drug effects on body weight.


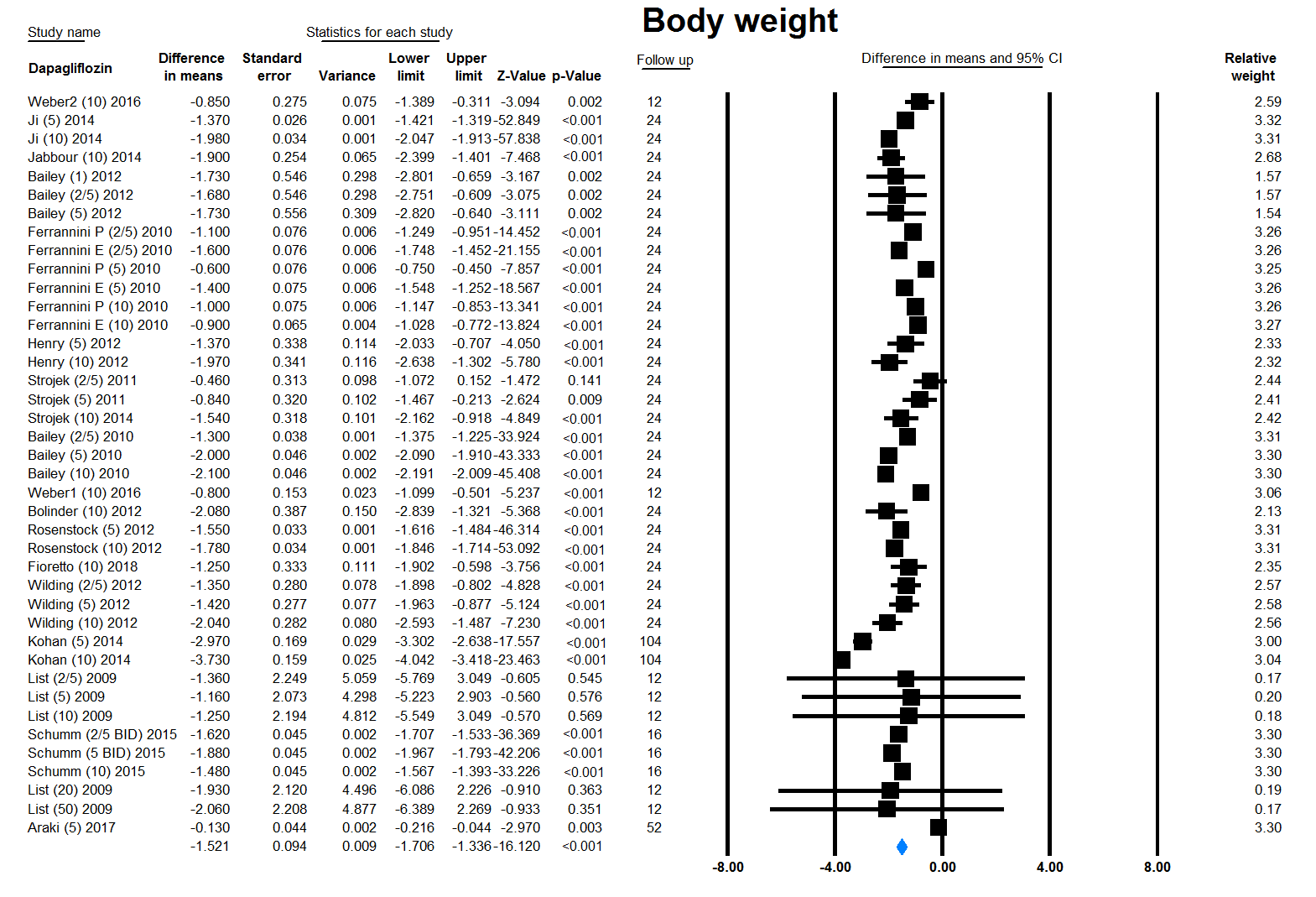


Figure S15: Meta-analysis of all empagliflozin studies to determine the drug effects on body weight.


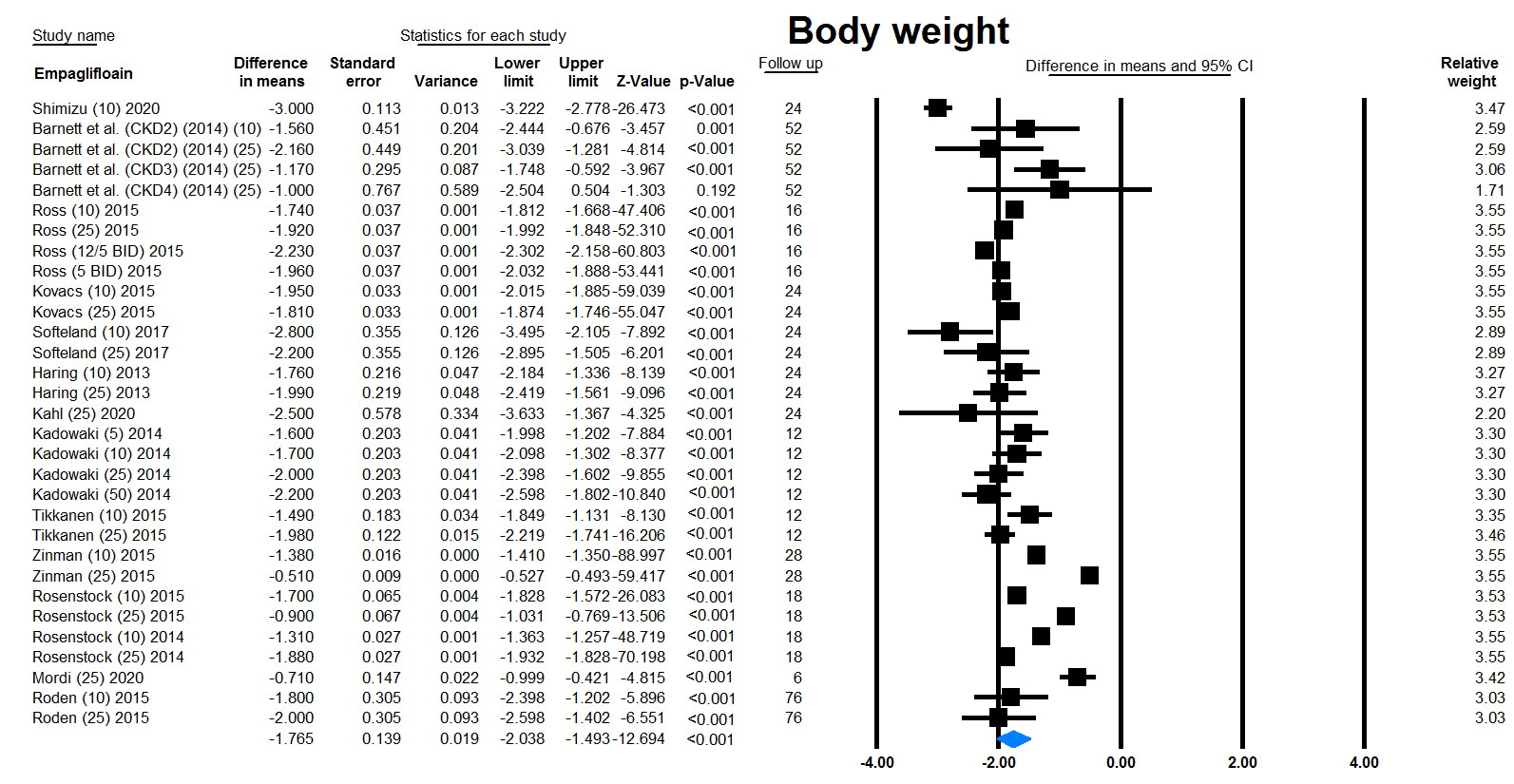

Supplement: Supplementary Materials — Table S1: search strategy. Figure S1: meta-analysis of all canagliflozin studies to determine the drug efficacy in serum uric acid reduction. Figure S2: meta-analysis of all dapagliflozin studies to determine the drug efficacy in serum uric acid reduction. Figure S3: meta-analysis of all empagliflozin studies to determine the drug efficacy in serum uric acid reduction. Figure S4: scatterplots of metaregression on canagliflozin variables (weeks of treatment, drug dosage, and duration of diabetes). Figure S5: scatterplots of metaregression on dapagliflozin variables (weeks of treatment, drug dosage, and duration of diabetes). Figure S6: scatterplots of metaregression on empagliflozin variables (weeks of treatment, drug dosage, and duration of diabetes). Figure S7: meta-analysis of all canagliflozin studies to determine the drug effects on HbA1c. Figure S8: meta-analysis of all dapagliflozin studies to determine the drug effects on HbA1c. Figure S9: meta-analysis of all empagliflozin studies to determine the drug effects on HbA1c. Figure S10: meta-analysis of all canagliflozin studies to determine the drug effects on FPG. Figure S11: meta-analysis of all dapagliflozin studies to determine the drug effects on FPG. Figure S12: meta-analysis of all empagliflozin studies to determine the drug effects on FPG. Figure S13: meta-analysis of all canagliflozin studies to determine the drug effects on body weight. Figure S14: meta-analysis of all dapagliflozin studies to determine the drug effects on body weight. Figure S15: meta-analysis of all empagliflozin studies to determine the drug effects on body weight. [file 7520632.f1.docx]
